# Contribution of *H. pylori* and Smoking Trends to US Incidence of Intestinal-Type Noncardia Gastric Adenocarcinoma: A Microsimulation Model

**DOI:** 10.1371/journal.pmed.1001451

**Published:** 2013-05-21

**Authors:** Jennifer M. Yeh, Chin Hur, Deb Schrag, Karen M. Kuntz, Majid Ezzati, Natasha Stout, Zachary Ward, Sue J. Goldie

**Affiliations:** 1 Center for Health Decision Science, Harvard School of Public Health, Boston, Massachusetts, United States of America; 2 Massachusetts General Hospital Institute for Technology Assessment, Boston, Massachusetts, United States of America; 3 Dana-Farber Cancer Institute, Harvard Medical School, Boston, Massachusetts, United States of America; 4 University of Minnesota School of Public Health, Minneapolis, Minnesota, United States of America; 5 Imperial College of London, London, England, United Kingdom; 6 Department of Population Medicine, Harvard Medical School and Harvard Pilgrim Health Care Institute, Boston, Massachusetts, United States of America; University of Southern California, United States of America

## Abstract

**Background:**

Although gastric cancer has declined dramatically in the US, the disease remains the second leading cause of cancer mortality worldwide. A better understanding of reasons for the decline can provide important insights into effective preventive strategies. We sought to estimate the contribution of risk factor trends on past and future intestinal-type noncardia gastric adenocarcinoma (NCGA) incidence.

**Methods and Findings:**

We developed a population-based microsimulation model of intestinal-type NCGA and calibrated it to US epidemiologic data on precancerous lesions and cancer. The model explicitly incorporated the impact of *Helicobacter pylori* and smoking on disease natural history, for which birth cohort-specific trends were derived from the National Health and Nutrition Examination Survey (NHANES) and National Health Interview Survey (NHIS). Between 1978 and 2008, the model estimated that intestinal-type NCGA incidence declined 60% from 11.0 to 4.4 per 100,000 men, <3% discrepancy from national statistics. *H. pylori* and smoking trends combined accounted for 47% (range = 30%–58%) of the observed decline. With no tobacco control, incidence would have declined only 56%, suggesting that lower smoking initiation and higher cessation rates observed after the 1960s accelerated the relative decline in cancer incidence by 7% (range = 0%–21%). With continued risk factor trends, incidence is projected to decline an additional 47% between 2008 and 2040, the majority of which will be attributable to *H. pylori* and smoking (81%; range = 61%–100%). Limitations include assuming all other risk factors influenced gastric carcinogenesis as one factor and restricting the analysis to men.

**Conclusions:**

Trends in modifiable risk factors explain a significant proportion of the decline of intestinal-type NCGA incidence in the US, and are projected to continue. Although past tobacco control efforts have hastened the decline, full benefits will take decades to be realized, and further discouragement of smoking and reduction of *H. pylori* should be priorities for gastric cancer control efforts.

*Please see later in the article for the Editors' Summary*

## Abstract 2

Jennifer Yeh and colleagues examine the contribution of *I*Helicobacter pylori*I* and smoking trends to the incidence of past and future intestinal-type noncardia gastric adenocarcinoma.

*Please see later in the article for the Editors' Summary*

## Editors' Summary

**Background:**

Cancer of the stomach (gastric cancer) is responsible for a tenth of all cancer deaths world-wide, with an estimated 700,000 people dying from this malignancy every year, making it the second most common cause of global cancer-related deaths after lung cancer. Unfortunately, the projected global burden of this disease estimate that deaths from gastric cancer will double by 2030. Gastric cancer has a poor prognosis with only a quarter of people with this type of cancer surviving more than five years. In order to reduce deaths, it is therefore of utmost importance to identify and reduce the modifiable risk factors associated with gastric cancer. Smoking and chronic gastric infection with the bacteria *Helicobacter pylori (H. pylori*), are known to be two common modifiable risk factors for gastric cancer, particularly for a type of gastric cancer called intestinal-type noncardia gastric adenocarcinoma (NCGA), which occurs at the distal end of the stomach and accounts for more than half of all cases of gastric cancer in US men.

**Why Was This Study Done?:**

*H. pylori* initiates a precancerous process, and so infection with this bacteria can increase intestinal-type NCGA risk by as much as 6-fold while smoking doubles cancer risk by advancing increasing progression of existing lesions. Changes in these two risk factors over the past century (especially following the US Surgeon General's Report on Smoking and Health in 1964) have led to a dramatic decline in the rates of gastric cancer in US men. Understanding the combined effects of underlying risk factor trends on health outcomes for intestinal-type NCGA at the population level can help to predict future cancer trends and burden in the US. So in this study, the researchers used a mathematical model to estimate the contribution of *H. pylori* and smoking trends on the decline in intestinal-type NCGA incidence in US men.

**What Did the Researchers Do and Find?:**

The researchers used birth cohorts derived from data in two national databases, the National Health and Nutrition Examination Survey (NHANES) and National Health Interview Survey (NHIS) to develop a population-based model of intestinal-type NCGA. To ensure model predictions were consistent with epidemiologic data, the researchers calibrated the model to data on cancer and precancerous lesions and using the model, projected population outcomes between 1978 and 2040 for a base-case scenario (in which all risk factor trends were allowed to vary over time). The researchers then evaluated alternative risk factors scenarios to provide insights on the potential benefit of past and future efforts to control gastric cancer.

Using these methods, the researchers estimated that the incidence of intestinal-type NCGA (standardized by age) fell from 11.0 to 4.4 per 100,000 men between 1978 and 2008, a drop of 60%. When the researchers incorporated only *H. pylori* prevalence and smoking trends into the model (both of which fell dramatically over the time period) they found that intestinal-type NCGA incidence fell by only 28% (from 12.7 to 9.2 per 100,000 men), suggesting that *H. pylori* and smoking trends are responsible for 47% of the observed decline. The researchers found that *H. pylori* trends alone were responsible for 43% of the decrease in cancer but smoking trends were responsible for only a 3% drop. The researchers also found evidence that after the 1960s, observed trends in lower smoking initiation and higher cessation accelerated the decline in intestinal-type NCGA incidence by 7%. Finally, the researchers found that intestinal-type NCGA incidence is projected to decline an additional 47% between 2008 and 2040 (4.4 to 2.3 per 100,000 men) with *H. pylori* and smoking trends accounting for more than 80% of the observed fall.

**What Do These Findings Mean?:**

These findings suggest that, combined with a fall in smoking rates, almost half of the observed fall in rates of intestinal-type NCGA cancer in US men between 1978 and 2008 was attributable to the decline in infection rates of *H. pylori*. Rates for this cancer are projected to continue to fall by 2040, with trends for both *H. pylori* infection and smoking accounting for more than 80% of the observed fall, highlighting the importance of the relationship between risk factors changes over time and achieving long-term reduction in cancer rates. This study is limited by the assumptions made in the model and in that it only examined one type of gastric cancer and excluded women. Nevertheless, this modeling study highlights that continued efforts to reduce rates of smoking and *H. pylori* infection will help to reduce rates of gastric cancer.

**Additional Information:**

Please access these Web sites via the online version of this summary at http://dx.doi.org/10.1371/journal.pmed.1001451.

- The National Cancer Institute gives detailed information about gastric cancer
- The Gastric Cancer Foundation has information on gastric cancer for patients and professionals
- Cancer Research UK explains types of gastric cancer

## Introduction

Gastric cancer (GC) is the second most common cause of cancer-related deaths worldwide, responsible for an estimated 700,000 deaths each year (10.4% of all cancer deaths) [1]. Based on current age-specific rates of GC and projected demographic changes, the annual number of expected deaths worldwide will increase to 1.4 million in 2030. Once diagnosed, the prognosis and treatment options are poor, with less than 27% surviving more than 5 y [2]. Reducing GC incidence through modification of risk factors may therefore be the most effective way to reduce GC mortality.

In the US, GC was the leading cause of cancer-related deaths among men in the early 1900s. While it has fallen dramatically since then, the precise reasons for the “unplanned triumph” are not well-established [3], though broadly attributed to improvements in living conditions and availability of refrigeration. The decline has been more pronounced for noncardia cancers, in particular intestinal-type tumors for which *H. pylori* infection is the leading risk factor [4]. Recent evidence suggests that cardia cancers may be increasing in frequency [5],[6]. Although histologic subtypes are sometimes difficult to distinguish, these trends in cancer incidence may suggest possible differences in tumor biology.

Intestinal-type noncardia gastric adenocarcinoma (NCGA), which accounts for over 50% of all GC cases in the US [7], develops through a series of relatively well-defined histological steps over several decades [8], and the influence of *H. pylori* and smoking influence on the carcinogenesis process have been well-described by epidemiologic studies [9]–[14]. By initiating the precancerous process, *H. pylori* infection increases intestinal-type NCGA risk by as much as 6-fold [10], while smoking elevates cancer risk by 2-fold by increasing progression risk of existing lesions to more advanced lesions [15]. As intestinal-type NCGA incidence has fallen over the past century, prevalence of both risk factors has also drastically changed. Only 33% of adults are currently infected with *H. pylori* [16], and after peaking to more than 50% in the 1955, smoking rates have declined to 20% since the 1964 publication of the first US Surgeon General's Report on Smoking and Health [17]–[19]. Other risk factors, including genetic factors and diet (e.g., low intake of fresh fruits and vegetables and/or high salt intake), are also important, although their effects on the gastric carcinogenesis are less well-understood.

Understanding the combined effects of underlying risk factor trends on population-level intestinal-type NCGA outcomes can help to predict future cancer trends and burden in the US and globally. While data on *H. pylori* prevalence and smoking rates in the US are available from the National Health and Nutrition Examination Survey (NHANES) [16] and National Health Interview Survey (NHIS) [18], these databases do not contain information on GC. Similarly, the Surveillance, Epidemiology and End Results (SEER) Program provides estimates of population-based cancer incidence, but lacks data on risk factors.

We employ a mathematical modeling framework capable of integrating available epidemiologic, clinical, and demographic data to understand the effect of risk factor trends on past and future population-level intestinal-type NCGA incidence rates among US men. Specifically, we aim to estimate the contribution of *H. pylori* and smoking trends on the decline in cancer incidence and explore the magnitude by which anti-smoking campaigns following the US Surgeon General's 1964 Report on Smoking and Health accelerated the decline.

## Methods

### Overview

**Figure 1:**
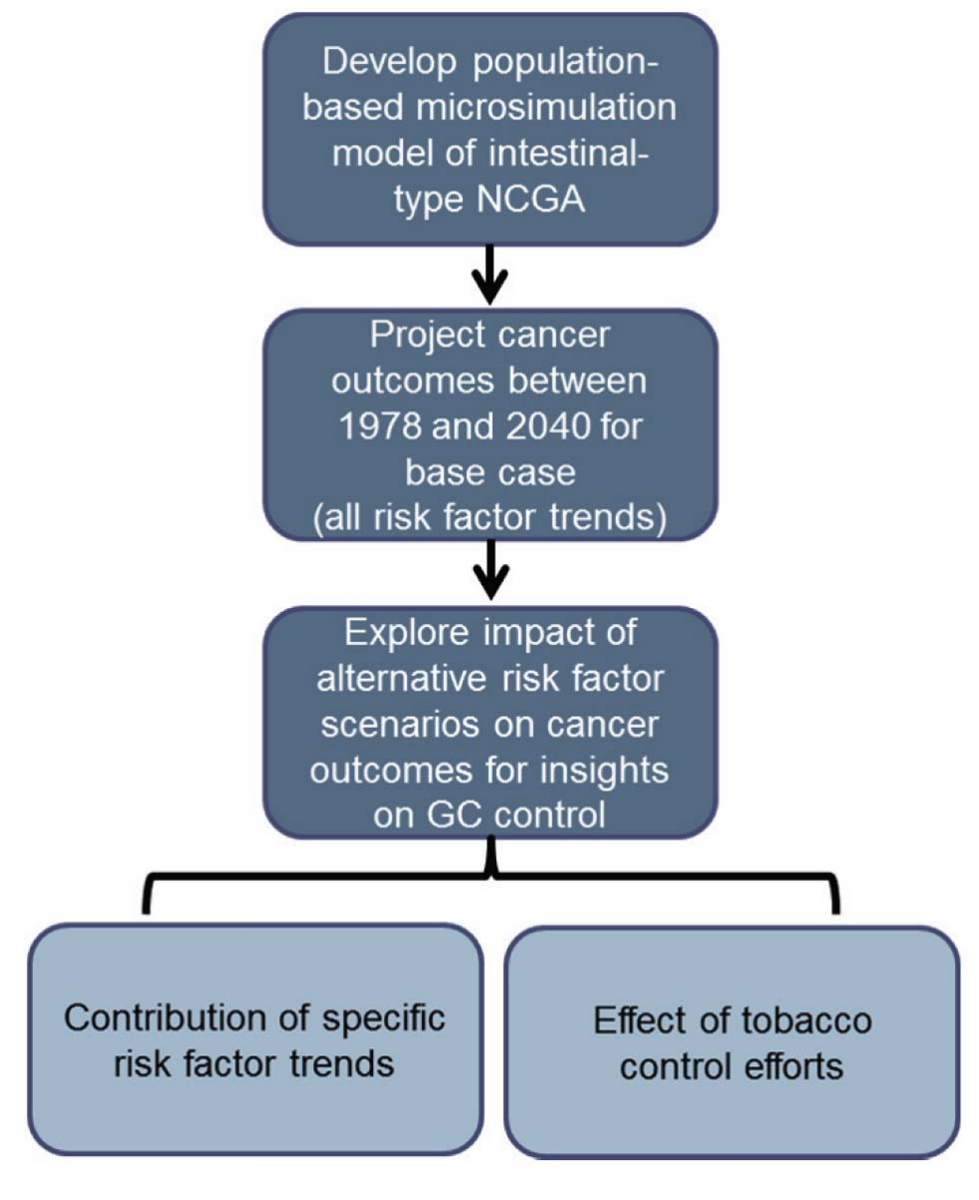
Overview of model-based approach. This figure depicts the three components of our trend analysis: (1) development of a population-based intestinal-type NCGA microsimulation model, which explicitly incorporates birth cohort-specific risk factor trends, background mortality rates, and population size, and identifies natural history progression rates via model calibration to US epidemiologic data; (2) projection of intestinal-type NCGA outcomes between 2008 and 2040 for the base case (all risk factor trends) using a subset of 50 randomly selected good-fitting parameter sets (identified via model calibration); and (3) estimation of intestinal-type NCGA outcomes under alternative risk factor or tobacco control scenarios for insights on GC control.

We developed a population-based microsimulation model of intestinal-type NCGA to estimate the impact of observed risk factor trends on past and future cancer incidence for US men (Figure 1). We focused on this subset of GCs, defined by criteria proposed and used by Lauren [20], Henson et al., [21], and Wu et al. [22], as the precancerous process and role of risk factors for this histologically distinct subgroup of distally located tumors has been well-established. The model explicitly incorporated the impact of *H. pylori* and smoking on disease natural history. To accomplish this, birth cohort trends were derived from NHANES and NHIS data, and the model was calibrated to US data on precancerous lesions and cancer to ensure model predictions were consistent with epidemiologic data. Using the model, we projected population-based intestinal-type NCGA outcomes between 1978 and 2040 in which all risk factor trends were allowed to vary over time (base-case scenario). We then used the model to evaluate alternative risk factor scenarios to provide insights on the potential benefit of past and future GC control efforts.

### Intestinal-Type NCGA Natural History Microsimulation Model

**Figure 2:**
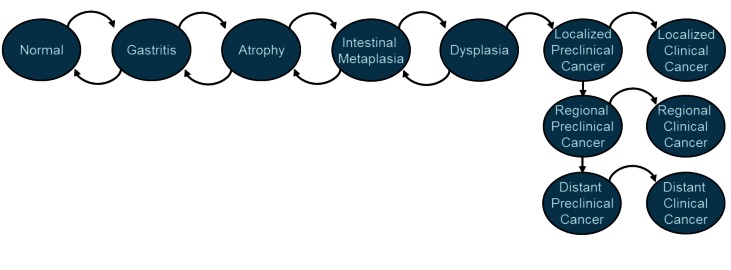
Intestinal-type NCGA model diagram. Intestinal-type NCGA develops through a series of precancerous health states as depicted. Each month, individuals face a risk of progression among the health states. Before invasive cancer develops, individuals can also regress to less advanced precancerous lesions. Individuals with preclinical (asymptomatic) cancer can remain asymptomatic or progress to symptomatic clinical cancer. Once individuals develop symptomatic cancer, they are assumed to receive treatment and do not progress to more advanced cancer states. All probabilities are constant, except for the age-specific transition from dysplasia to preclinical cancer.

To allow for greater flexibility in capturing multiple dimensions of heterogeneity, we refined a previously described intestinal-type NCGA natural history model [23] to function as a microsimulation model that simulates individuals within multiple 5-y birth cohorts (born between 1881 and 2025) to generate population-based cancer outcomes for men between 1978 and 2040. In the model, intestinal-type NCGA develops through a series of precancerous lesions, which may progress to dysplasia and eventually invasive cancer (Figure 2). At the start of the simulation, 20-y-old individuals are assigned a birth cohort-specific risk factor profile and enter the model. Each month, using rates derived via previously described model calibration methods (see Table 1 and Text S1) [23], individuals face a risk of progression among the health states. On the basis of epidemiologic studies, we assumed that in addition to causing gastritis, *H. pylori* increases the risk of atrophy [9], while smoking increases the risk of progression to intestinal metaplasia and dysplasia [11],[12].

**Table 1:** Intestinal-type NCGA model parameter values.

Parameter^a^	Range Identified Via Model Calibration
**Natural history**	
Gastritis to atrophy	0.0001
Atrophy to intestinal metaplasia	0.0048–0.0200
Intestinal metaplasia to dysplasia	0.0005–0.0010
Dysplasia to preclinical cancer	
Age 20–29	0.000000–0.000005
Age 30–39	0.000000–0.000009
Age 40–49	0.000004–0.00003
Age 50–59	0.0002–0.0003
Age 60–69	0.0004–0.0008
Age 70–79	0.0007–0.0018
Age 80–89	0.0011–0.0034
Age 90+	0.0016–0.0066
Preclinical to clinical cancer	0.0410–0.0830
Dysplasia to intestinal metaplasia	0.0051–0.0090
Intestinal metaplasia to atrophy	0.0001–0.0086
Atrophy to gastritis	0.0005–0.0099
**Clinical cancer detection**	
Local	0.006–0.014
Regional	0.024–0.056
Distant	0.034–0.400
**Risk factors on disease progression (relative risk)**	
*H. pylori*	
Gastritis to atrophy	16.1–41.3
Smoking	
Atrophy to intestinal metaplasia^b^	
<20 cigarettes per day	1.1–3.7
≥20 cigarettes per day	3.6–17.0
Former smoker	1.1–3.7
Intestinal metaplasia to dysplasia^b^	
<10 cigarettes per day	1.0–2.6
≥10 cigarettes per day	2.1–2.8
Former smoker	1.0–2.6
All other risk factors	
Gastritis to atrophy^c^	0.039–0.098

a Monthly probabilities unless otherwise noted.

b Relative to non-smokers (RR = 1).

c Constant exponential rate (r) of decline per birth cohort as described in the following equation: (1−r)∧t, where t = year of birth − 1901.

**Figure 3:**
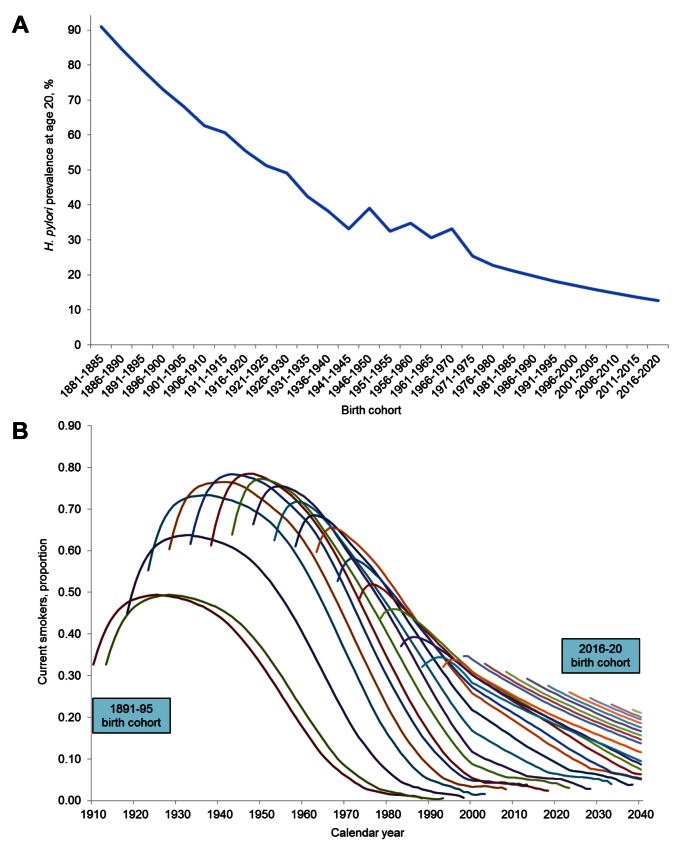
Birth cohort risk factor profiles. (A) *H. pylori* prevalence at age 20 for birth cohorts between 1881 and 2020 based on NHANES III and Continuous NHANES (1999–2000) data. For pre-1900 and post-1980 birth cohorts for which data were unavailable, we extrapolated the observed 7.1% rate of decline between successive 5-y birth cohorts between the 1901–1905 and 1976–1980. (B) The proportion of current smokers by 5-y birth cohorts between 1891 and 2020 (starting at age 20). For birth cohorts born after 1983 (and cohorts with individuals still alive past calendar year 2000) for which data from the NHIS-based Smoking History Generator were unavailable, we extrapolated observed trends and extended profiles until all individuals either died or reached 100 y of age. Specifically, for post-1983 birth cohorts, we assumed that smoking rates peaked at age 20, initiation rates declined by 5.7% every 5 birth cohort years, and the annual risk of cessation remained constant at 2% [54],[55].

For each individual, we assigned *H. pylori* status at age 20 based on NHANES data (Figure 3). As *H. pylori* infection is acquired in childhood and persists unless treated with antibiotics [24],[25], we assumed that an individual's status remained unchanged throughout their lifetime. Smoking profiles for each individual, including initiation and cessation age, were derived from the NHIS-based Smoking History Generator (SHG) developed by the National Cancer Institute (NCI) Cancer Intervention and Surveillance Modeling Network (CISNET) (Figure 3) [26],[27]. As the impact of smoking on disease progression varies by intensity [11],[12], we categorized smokers into four subgroups: low (<10 cigarettes per day [CPD]), moderate (10–19 CPD), heavy (≥20 CPD), and former smokers. The effect of smoking cessation on disease progression was also assumed to be immediate upon quitting, with progression rates declining and remaining at former smoker levels.

Given the multifactorial etiology of intestinal-type NCGA, factors other than *H. pylori* and smoking have contributed to the observed decline in cancer incidence. As such, we allowed birth cohort-specific disease risk to vary over time, an approach commonly used in population-based microsimulation models [28]. Specifically, as multi-stage models of gastric carcinogenesis suggest exposures early on are likely responsible for incidence declines among successive birth cohorts [3],[8],[29], we operationalized the “all other risk factors” effect as a constant exponential rate of decline on the risk of developing atrophy beginning with the 1901 birth cohort.

To reflect changes in all-cause background mortality, we used birth cohort-specific life tables obtained from the Berkeley Mortality Database (http://www.demog.berkeley.edu/~bmd/) and US Social Security Administration [30], and adjusted for smoking intensity using relative risk estimates from the American Cancer Society Cancer Prevention Study II (CPS-II) (see Text S1) [31].

### Model Outcomes and Uncertainty Analysis

Model outcomes included age-standardized incidence, number of annual cases, and number of total cases for intestinal-type NCGA. For age-standardized cancer incidence, we used the US 2000 standard population for comparability to SEER estimates (assuming cancer risk among individuals less than 20 y old was negligible) [32]. For annual number of cases, we incorporated population size estimates from the US Census Bureau into the model to reflect the impact of population growth and ageing on cancer cases [33]. The model also projected the prevalence of precancerous lesions among individuals 20 y and older.

To reflect the impact of disease natural history uncertainty on modeled outcomes, using previously described calibration methods [23], we identified multiple parameter sets that fit equally well to epidemiologic data on age-specific precancerous lesions prevalence [34] and GC incidence [7]. Among the good-fitting parameter sets identified, we randomly selected a subset of 50 parameter sets for this analysis and report the mean and range (minimum, maximum) for all model outcomes. See Text S1 for additional details on model development and calibration.

### Intestinal-Type NCGA Projections between 1978 and 2040: Scenarios

For our base-case scenario, we incorporated all observed risk factor trends and projected intestinal-type NCGA outcomes over two time periods: 1978–2008 and 2008–2040. To explore the impact of alternative risk factor scenarios, we re-ran the model for several other risk factor scenarios and compared model outcomes with the base-case scenario. First, to estimate the combined contribution of *H. pylori* and smoking trends on cancer outcomes over time, we allowed only *H. pylori* and smoking trends to vary (e.g., “all other risk factors” halted at pre-1900 levels). We also estimated the contribution of individual risk factors by allowing each risk factor to vary (i.e., *H. pylori*) while holding the rates of the other two risk factors (i.e., smoking patterns and “all other risk factors”) constant at pre-1900 birth cohort levels.

For insight on the relative impact of the lower smoking initiation and higher cessation rates that were observed after the 1964 Report on Smoking and Health [19], we evaluated two additional hypothetical scenarios, previously described in the context of lung cancer [28],[35]: (1) no tobacco control, in which mid-century tobacco control efforts were assumed to have never been implemented, and (2) complete tobacco control, in which all smoking ceased abruptly in 1965, with all smokers quitting permanently in 1965 and no smoking initiation after 1964.

For each scenario, we calculated the percent decline in cancer incidence over two time periods: 1978–2008 and 2008–2040. To understand the effects of risk factors and tobacco control, we also report the relative contribution of alternative risk factor scenarios to the base-case scenario decline in cancer incidence (defined as the risk factor scenario percent decline divided by base-case scenario percent decline, and reported as a percentage).

## Results

### Age-Standardized Intestinal-Type NCGA Incidence

#### Historical trends, 1978 to 2008

For our base case, reflecting all observed risk trends, the model estimated that the age-standardized intestinal-type NCGA incidence declined from 11.0 to 4.4 per 100,000 men between 1978 and 2008 (60% decline), less than a 3% discrepancy with SEER estimates for that period (Table 2) [7].

**Table 2:** Modeled intestinal-type NCGA outcomes between 1978 and 2040: age-standardized incidence, percent change in incidence, and relative contribution of alternative risk factor scenarios to the base-case scenario percent decline in incidence.

Scenarios^a^^,^ b ^,^ c	Age-Standardized Intestinal-Type NCGA Incidence (per 100,000)			Percent Change in Age-Standardized Intestinal-Type NCGA Incidence, Mean (Range)^d^		Percent Relative Contribution of Alternative Risk Factor Scenario to Base-Case Scenario Percent Decline in Age-Standardized Intestinal-Type NCGA Incidence, Mean (Range)^d^^,^ e	
				Historical	Projected	Historical	Projected
	1978	2008	2040	1978–2008	2008–2040	1978–2008	2008–2040
Base case (all risk factors)	11.0	4.4	2.3	−60.1 (−55.5 to −64.8)	−47.3 (−34.7 to −59.4)	—	—
*H. pylori* and smoking only	12.7	9.2	5.7	−28.1 (−17.8 to −35.4)	−37.7 (−27.0 to −47.7)	46.7 (29.8–57.9)	80.5 (60.7–100.0)^f^
*H. pylori* only	10.9	8.1	6.4	−25.7 (−21.1 to −29.7)	−21.4 (−16.5 to −25.4)	42.8 (35.4–48.4)	46.0 (34.7–65.8)
Smoking only	15.4	15.1	12.1	−2.0 (−10.3 to +6.6)	−19.9 (−10.6 to −29.2)	3.2 (0.0–16.8)^g^	41.8 (28.6–66.6)
All other causes only	11.1	5.7	4.6	−49.0 (−40.0 to −55.9)	−19.2 (−6.1 to −33.4)	81.5 (70.3–93.4)	40.2 (11.9–68.8)
+*H. pylori*	9.6	3.7	2.6	−61.2 (−55.2 to −67.8)	−30.4 (−18.2 to −43.3)	101.9 (91.9–112.9)	64.4 (39.9–81.1)
+Smoking	12.9	6.5	4.0	−49.6 (−43.9 to −54.5)	−38.3 (−24.9 to −53.6)	82.6 (74.5–89.7)	80.5 (62.6–98.2)
No tobacco control	11.4	5.0	3.5	−56.0 (−49.8 to −59.8)	−30.4 (−17.0 to −43.0)	93.1 (82.7–100.0)^h^	64.3 (46.1–83.4)
Complete tobacco control	10.1	3.6	1.6	−63.9 (−57.7 to −67.8)	−55.0 (−37.2 to −76.6)	106.3 (100.0–114.2)^i^	115.8 (100.0–140.7)^i^

a For scenarios in which smoking was halted at pre-1900 birth cohort levels, we classified all individuals who initiated smoking after 1925 as never smokers (as smoking rates were relatively stable before then) and assumed that all smokers faced an annual rate of cessation of 1%.

b For scenarios in which *H. pylori* was halted at pre-1900 birth cohort levels, we assumed a prevalence of 73%.

c For scenarios in which “all other risk factors” was halted at pre-1900 birth cohort levels, we assumed a negligible rate of decline in the probability of developing atrophy for all birth cohorts.

d Among the 50 randomly selected good-fitting natural history parameter sets identified via calibration.

e Based on comparisons to the base-case scenario within each parameter set. Calculated as the alternative risk factor scenario percent decline divided by the base-case scenario percent decline, and reported as a percentage.

f Range includes estimates for two parameter sets in which the intestinal-type NCGA decline estimated with only *H. pylori* and smoking trends exceeded the base case, reflecting the underlying dynamics with “all other risk factors.” Assumed the percentage of the base-case scenario explained by “*H. pylori* and smoking only” was 100% for these parameter sets.

g Range includes 14 parameter sets for which intestinal-type NCGA incidence increased as a result of smoking trends. Assumed the percentage of the base-case scenario explained by “smoking alone” was zero for these parameter sets.

h Range includes estimates for two parameter sets in which more than 100% of the observed decline was explained by “no tobacco control,” as a result of higher background mortality rates among smokers (e.g., intestinal-type NCGA incidence was lower as higher smoking rates resulted in a greater number of individuals dying from smoking-related competing risks and therefore fewer individuals being at risk to develop intestinal-type NCGA). Assumed the percentage of the base-case scenario explained by “no tobacco control” was zero for these parameter sets.

i Range includes estimates for six parameter sets (two for 1978–2008 and four for 2008–2040) in which less than 100% of observed decline was explained by “complete tobacco control,” as a result of lower background mortality rates among non-smokers (e.g., intestinal-type NCGA incidence was higher as lower rates of smoking resulted in more individuals living longer and developing intestinal-type NCGA). Assumed the percentage of the base-case scenario explained by “complete tobacco control” was 100% for these parameter sets.

**Figure 4:**
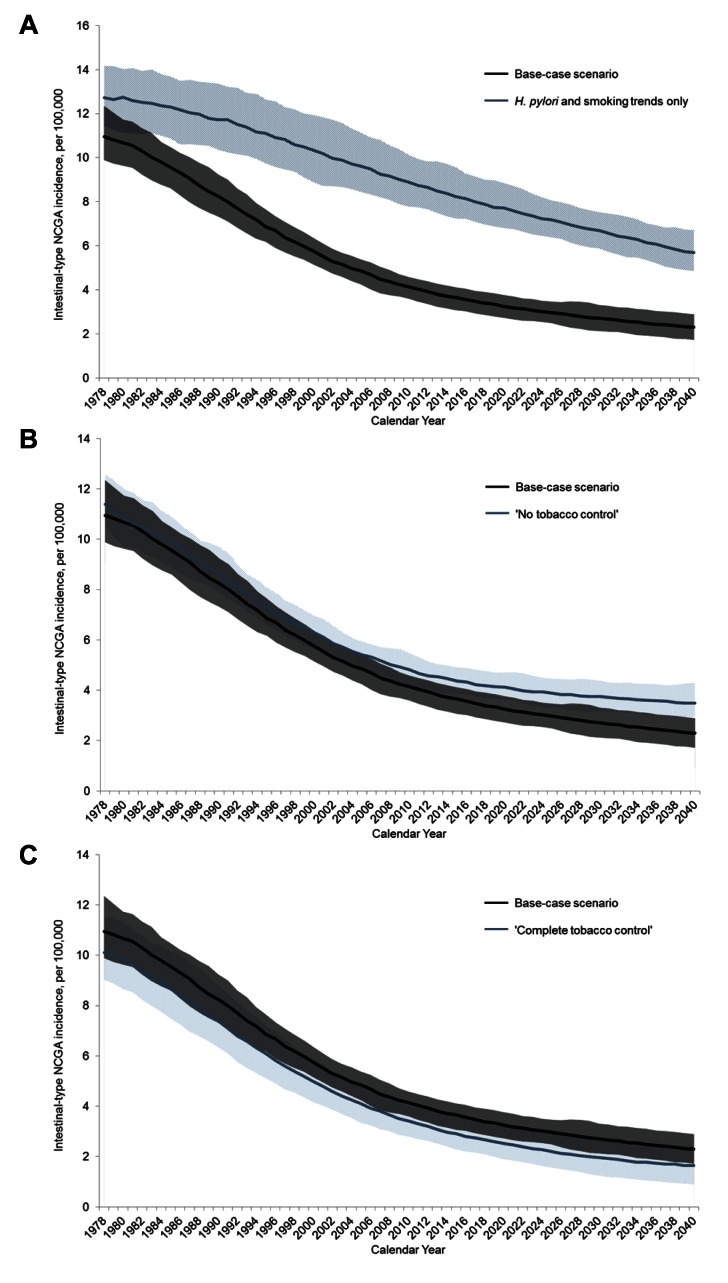
Modeled age-standardized intestinal-type NCGA incidence between 1978 and 2040 for select scenarios. (A–C) Age-standardized intestinal-type NCGA incidence for select scenarios. Mean (solid line) and range (shaded area) among the 50 randomly selected good-fitting parameter sets are shown. (A) Cancer incidence for the base-case scenario (all risk factor trends) (black) and *H. pylori* and smoking only scenario (light blue). (B) Cancer incidence for the base-case scenario (black) and “no tobacco control” scenario (light blue). (C) Cancer incidence for the base-case scenario (black) and “complete tobacco control” scenario (light blue). Ranges were calculated across all 50 parameter sets and smoothed with a 3-y moving average.

When only observed *H. pylori* prevalence and smoking trends were incorporated in the model (e.g., “all other risk factors” held constant at pre-1900 birth cohort levels), intestinal-type NCGA incidence decreased by 28% (12.7 to 9.2 per 100,000 men). This suggests that *H. pylori* and smoking trends are responsible for 47% (range = 30%–58%) of the observed cancer decline (Figure 4). Evaluated individually, *H. pylori* (43%; range = 35%–48%) and “all other risk factors” (82%; range = 70%–93%) were responsible for a significant proportion of the cancer decline; in contrast, smoking was responsible for a much smaller fraction (3%; range = 0%–17%). Among the 50 good-fitting parameter sets, the proportion attributable to “all other risk factors” was consistently greater than for *H. pylori*, and the proportion for *H. pylori* was greater than for smoking.

Under the “no tobacco control” scenario, the model estimated that age-standardized intestinal-type NCGA incidence declined 56% (11.4 to 5.0 per 100,000 men) (Figure 4). This finding suggests that the lower smoking initiation and higher cessation rates observed after the 1960s accelerated the relative decline in intestinal-type NCGA incidence by 7% (calculated as the base case percent decline divided by the “no tobacco control” percent decline = 107.4); the relative decline ranged from 0% to 21% among the 50 good-fitting parameter sets. Under the “complete tobacco control” scenario, compared with the base case, the relative decline in intestinal-type NCGA incidence would have been greater by 6% (range = 0%–14%) (Figure 4).

#### Projected trends, 2008 to 2040

For the base case, with continuation of risk factor trends, intestinal-type NCGA incidence is projected to decline an additional 47% between 2008 and 2040 (4.4 to 2.3 per 100,000 men) (Table 2). During this period, *H. pylori* and smoking trends would account for 81% (range = 61%–100%) of the observed cancer incidence decline. Evaluated separately, trends in *H. pylori* (46%; range = 35%–66%), smoking (42%; range = 29%–67%), and “all other risk factors” (40%; range = 12%–69%) would each account for a substantial proportion of the projected decline. *H. pylori* trends were responsible for a greater proportion of the observed decline in 22 of the 50 good-fitting parameter sets (44%) than either of the other two risk factors.

Under the “no tobacco control” scenario, the model projected that between 2008 and 2040, intestinal-type NCGA incidence would decline 30% (5.0 to 3.5 per 100,000 men). This implies that during this time period, smoking behavior changes beginning in the 1960s would accelerate the relative decline in intestinal-type NCGA incidence by 56% (range = 20%–117%) (as compared to 7% [range = 0%–21%] between 1978 and 2008). Similarly, had “complete tobacco control” occurred in 1965, the relative decline in intestinal-type NCGA incidence between 2008 and 2040 would also be greater by 16% (range = 0%–41%) (versus 6% [range = 0%–14%] between 1978 and 2008).

### Number of Intestinal-Type NCGA Cases

Reflecting the combined effects of changing risk factor trends and population growth and ageing on cancer outcomes (in contrast to age-standardized cancer incidence), the model estimated that between 1978 and 2008, the annual number of intestinal-type NCGA cases for the base case fell by 33% (6,180 to 4,160 cases) (range = 24%–41%) (Table 3). Between 2008 and 2040, the number of cases continued to decline by 10% (to 3,760 cases). As a result of the uncertainty in the combined effects of continued risk factor trends and population growth, the percent change among the 50 good-fitting parameter sets ranged from a 31% decline to a 12% increase during this period.

**Table 3:** Modeled intestinal-type NCGA outcomes between 1978 and 2040: annual number of cancer cases and percent change in number of cases.

Scenarios	Annual Number of Intestinal-Type NCGA Cases			Percent Change in Annual Number of Intestinal-Type NCGA Cases, Mean (Range)^a^	
				Historical	Projected^b^
	1978	2008	2040	1978–2008	2008–2040
Base case (all risk factors)	6,180	4,160	3,760	−32.7 (−23.7 to −40.8)	−9.8 (−30.9 to 12.4)
*H. pylori* and smoking only	7,510	8,770	9,050	16.8 (4.9 to 30.0)	3.3 (−12.7 to 22.2)
No tobacco control	6,380	4,450	4,960	−30.3 (−20.9 to −38.1)	11.7 (−7.9 to 29.6)
Complete tobacco control	5,940	3,910	3,050	−34.2 (−25.1 to −41.8)	−22.0 (−58.3 to 8.6)

a Among the 50 randomly selected good-fitting natural history parameter sets identified via calibration.

b Range for percent change included both positive and negative estimates, reflecting the uncertainty of the combined effects of continued risk factor trends and population growth on annual intestinal-type NCGA cases.

**Figure 5:**
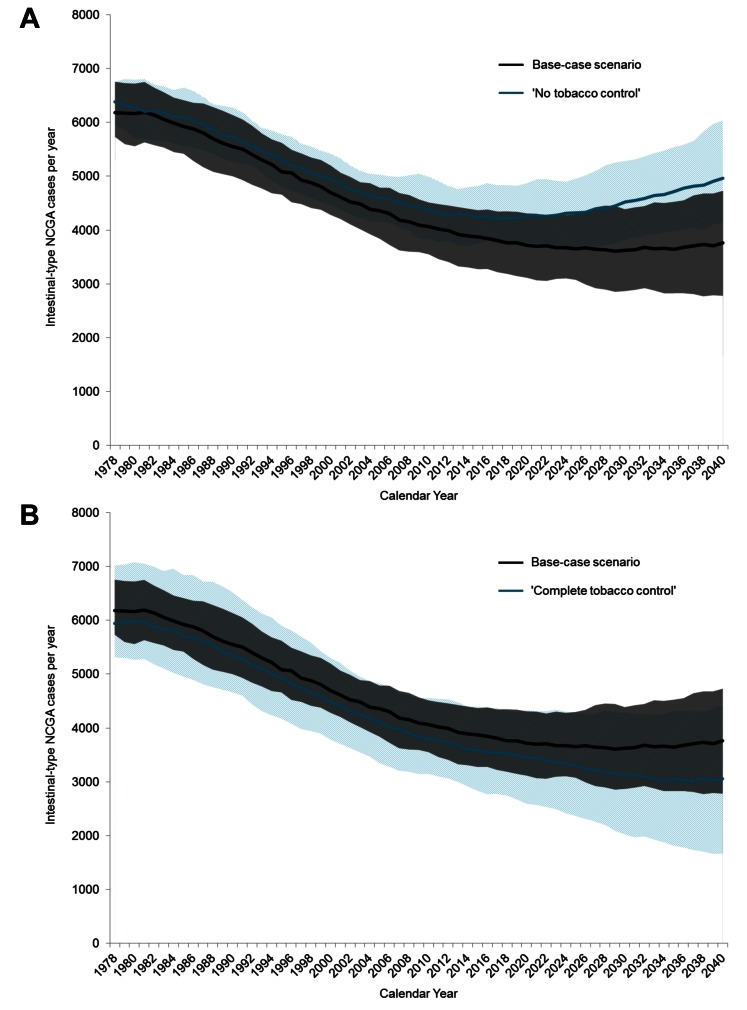
Modeled annual intestinal-type NCGA cases between 1978 and 2040 for select scenarios. These figures show the number of annual incident intestinal-type NCGC cases. Mean (solid line) and range (shaded area) among the 50 randomly selected good-fitting parameter sets are shown. (A) Cancer cases for the base-case scenario (black) and “no tobacco control” scenario (light blue). (B) Cancer cases for the base-case scenario (black) and “complete tobacco control” scenario (light blue). Ranges were calculated across all 50 parameter sets and smoothed with a 3-y moving average.

Under the “no tobacco control” scenario, the relative annual number of intestinal-type NCGA cases decreased by 30% (range = 21%–38%) between 1978 and 2008, and by 12% between 2008 and 2040 (Figure 5). Among the 50 good-fitting parameter sets, there was considerable uncertainty in the 2008–2040 estimates, ranging from a relative 8% decrease to a relative 30% increase in annual number of cases. Compared to base-case estimates, this suggests that anti-smoking efforts averted a total of 5,770 cases (3%; range = 0%–7%) between 1978 and 2008, and potentially as many as 22,470 cases (15%; range = 6%–23%) between 2008 and 2040. Had “complete tobacco control” in 1965 been possible, compared to the base-case scenario, an additional 19,890 cases (7%; range = 0.2%–13%) could have been potentially averted during the overall 1978 to 2040 time period (Figure 5).

### Prevalence of Precancerous Gastric Lesions

**Figure 6:**
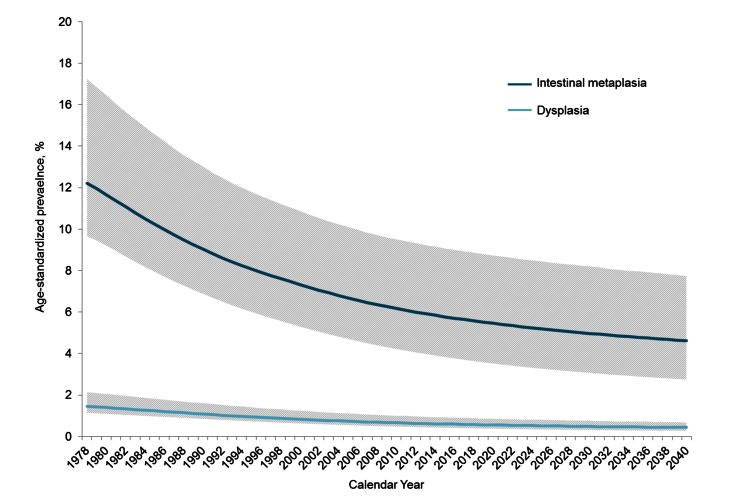
Age-standardized prevalence of precancerous lesions between 1978 and 2040. For individuals 20 y and older, age-standardized prevalence of intestinal metaplasia (dark blue) and dysplasia (light blue) are shown in this graph. Mean (solid line) and range (shaded region) among the 50 randomly selected good fitting parameter sets are shown to reflect the impact of natural history uncertainty on prevalence estimates.

Between 1978 and 2008, the model estimated that age-standardized prevalence of intestinal metaplasia and dysplasia both declined by approximately 50% between 1978 and 2008 (intestinal metaplasia range = 41%–56%; dysplasia range = 48%–58%). The decline is projected to continue, though at slower rates between 2008 and 2040 (intestinal metaplasia range = 20%–39%; dysplasia range = 30%–46%) (Figure 6).

## Discussion

The US decline in GC has been remarkable over the past century. While *H. pylori* is estimated to be responsible for the majority of distal tumors [36], the effect of declining childhood infection rates on intestinal-type NCGA over time has not been previously estimated. Incorporating the best available epidemiologic, etiologic, and demographic data, our microsimulation model analysis suggests that, in combination with changes in smoking patterns, nearly 50% of the observed cancer decline between 1978 and 2008 is attributable to the decline in *H. pylori* infection rates. Further, the decline in cancer incidence is projected to continue through 2040, with the contribution of *H. pylori* and smoking increasing to 80%. As with intestinal-type NCGA risk, the absolute number of cancer cases has also declined each year, though at a projected slower rate between 2008 and 2040 due to the combined effects of continued risk factor trends and demographic changes.

Our model-based findings highlight the challenges and successes of prevention efforts for GC. We found that a significant time lag exists between the implementation of any risk factor modification and population-level effects, even those that target progression rates of advanced lesions. For example, our model estimated that following the US Surgeon General's first report on the consequences of smoking, tobacco control efforts beginning in the mid-1960s accelerated the decline in intestinal-type NCGA incidence by only 7% between 1978 and 2008. However, in the following 22-y period, the decline may increase by 8-fold to 56%. In addition, we estimate that tobacco control efforts will avert 9% (28,000 cases) of intestinal-type NCGA cases between 1978 and 2040. In conjunction with the estimated 34% of lung cancer deaths averted among men between 1975 and 2000 [28], our estimates for GC provide further evidence of the health benefits of tobacco control.

Several other important insights can be gained from our findings. First, compared to smoking, the contribution of *H. pylori* on the decline in intestinal-type NCGA was several-fold greater between 1978 and 2008, but equal in magnitude between 2008 and 2040, reflecting the differential time trends for each risk factor. *H. pylori* prevalence began to decline in the early 1900s while smoking rates increased until the mid-1950s and declined in the following decades. In addition, the sum of the individual contributions for *H. pylori* and smoking exceeded the decline estimated with both concurrent trends. This illustrates the underlying risk factor dynamics on gastric carcinogenesis. Because *H. pylori* affects disease progression early on, and smoking at more advanced stages, as *H. pylori* prevalence declines, fewer people develop advanced lesions and benefit from lower smoking rates. As such, reductions in these two risk factors do not appear to be additive in their effects on cancer incidence, which is consistent with each risk factor influencing a different but sequential step in gastric carcinogenesis. These findings could have significant implications for GC control strategies.

Second, we attempted to calibrate our model by incorporating only *H. pylori* and smoking trends, but were unable to fit to the decline observed in SEER between 1978 and 2008 until we allowed the probability of developing atrophy to decline exponentially among consecutive 5-y birth cohorts. This finding suggests that changes in other risk factors are needed to explain the decline in intestinal-type NCGA incidence over time, and, according to our model, specifically risk factors that changed substantially during the first half of the 20th century. Availability of refrigeration may be one of these risk factors. Rates of US refrigeration rose steadily during the early 20th century [3], and data from Japan suggest that GC mortality rates declined as the proportion of households with refrigeration increased [37]. The underlying mechanism of refrigeration on intestinal-type NCGA risk likely stems from reduced salt consumption, and possibly increased intake of fresh fruits and vegetables [38]. Consistent with our findings, clinical studies suggest that the salt intake may act synergistically with *H. pylori* to promote the development of GC [39]–[42]. In addition, our model suggests that *H. pylori* is largely responsible for the progression of gastritis to atrophy (relative risk ranged from 16 to 41 among the good-fitting natural history parameter sets identified via model calibration).

Other risk factors that may explain the observed decline in intestinal-type NCGA include genetic profiles, virulence of *H. pylori* strain, and bacterial/host genotypic features [43]. However, these factors are not likely to have dramatically changed over time. Use of histamine-2-reception antagonists and proton pump inhibitors, the most common treatment for gastric acid production reduction, has changed over time. Yet evidence on the associated intestinal-type NCGA risk for these pharmacological treatments is inconclusive [44]. In addition, with both not having been introduced until the 1970s or 1980s, they are unlikely to explain the decrease in risk that our model suggestions should have started in the early 1900s.

Third, our model demonstrates the importance of considering the temporal relationships between risk factor changes and the attainment of long-term benefits on cancer outcomes. As the decline in *H. pylori* infection rates (albeit at different rates) and the tobacco epidemic are global phenomena, and the multifactorial etiology of intestinal-type NCGA is largely driven by these two risk factors, our findings provide important insight on the potential expected declines in other countries and may be in particular generalizable for countries experiencing similar risk factor trends. Because of the variability in risk factor trend magnitude and timing, the contribution of *H. pylori* and smoking to the decline of cancer incidence will depend on country-specific trends and the underlying temporal relationship between the risk factors. For example, countries experiencing declines in *H. pylori* prevalence during the initial stages of the tobacco epidemic (where uptake of smoking has not yet plateaued) will likely have smaller reductions in cancer outcomes than countries that are undergoing declines in both risk factors at the same time. The influence of other risk factor trends in each country will be an important consideration as well.

Fourth, recent trend analyses suggest that while age-standardized NCGA rates are declining overall [6],[22],[45]–[47], age-specific rates may be increasing [6],[45]. Our analyses found that among younger individuals, age-specific intestinal-type NCGA rates have declined and are projected to continue to decline because of *H. pylori*, smoking, and other risk factor trends. This was true even in the “no tobacco control” scenario (see Text S1). The increase is likely not related to changes (or rather lack of changes) in smoking behaviors. We also found that along with age-standardized intestinal-type NCGA incidence, prevalence of intestinal metaplasia and dysplasia has significantly declined over time. This is relevant from a clinical standpoint as the rise in gastroesophageal reflux disease (GERD) and endoscopy use has resulted in detection of incidental precancerous lesions. Our model suggests that with continued risk factor trends, between 2008 and 2040, the prevalence will not vary greatly for intestinal metaplasia (4%–6%) or dysplasia (<1%), and are similar to estimates for the Netherlands (9.5% and 0.7% in 2004).

While simulation modeling has been used to better understand the impact of lifestyle changes and cancer control measures on colorectal [48], lung [28], and breast cancer [49] incidence and mortality, our approach has several limitations. First, we assumed all other risk factors influenced gastric carcinogenesis as one factor early on in the precancerous process. Other environmental factors, or rather a combination, may in reality have affected the probability of developing atrophy and/or other disease transitions over time. Smoking may have affected gastric carcinogenesis at other points as well. As data on additional risk factors and their impact on precancerous process become available, our model can be updated and revised to reflect these changes. In particular, incorporating salt intake or other dietary risk factors into the model may help to further understand the contribution of risk factor changes to the decline in cancer incidence. We focused only on intestinal-type NCGA, and *H. pylori* and smoking affect the risk for other types of GC, including mucosa-associated lymphoid tissue (MALT) lymphomas [50]. However, a strength of our study is to look specifically at trends in noncardia intestinal gastric adenocarcinoma, as previous descriptive studies of SEER have been unable to examine trends by individual subsite or by Lauren classification due to incomplete reporting and small numbers [45].

In addition, our natural history parameters are based on model calibration, and are surrounded by uncertainty. We sought to explicitly capture the impact of natural history uncertainty on modeled outcomes by using a random subset of 50 good-fitting parameter sets to provide upper and lower bounds of results. Our model-based approach required several simplifying assumptions, such as assuming the effect of smoking cessation on disease progression rates was immediate or that the relative risk associated with smoking for background mortality was constant across birth cohorts, but were based on the best data available [11],[12],[31]. Birth cohort risk factor trends were estimated from national surveys, which may be biased and underestimate the true prevalence of *H. pylori* or smoking. For example, we also did not account for changes in *H. pylori* treatment and/or endoscopy rates, which may have caused us to underestimate the proportion of intestinal-type NCGA incidence attributable to changes in *H. pylori* prevalence.

We also only focused on men. While the decline in *H. pylori* prevalence has been similar, the smoking epidemic among women has been quite distinct from men [51],[52]. Absolute intestinal-type NCGA risk is also lower among women [2]. As such, our results likely provide an upper bound estimate on the proportion of intestinal-type NCGA incidence among women attributable to declining *H. pylori* and smoking trends; specific analyses are needed to provide more accurate estimates for women and the overall population. In addition, our projections may not fully account for the impact of projected rates of immigration of individuals from high GC risk countries. As GC risk is largely determined by exposures early on in life, cancer projections may be especially sensitive to the increasing proportion of individuals who migrate to the US after childhood, particularly if their risk factor profiles differ from the average NHANES and NHIS patterns on which our model was based. NHANES and NHIS data also suggest that *H. pylori* [53] and smoking trends [51] have varied by race/ethnicity, and it is unclear whether risk factor trends have widened or narrowed observed disparities in intestinal-type NCGA risk. As such, next steps in our efforts to provide a more comprehensive understanding of the US decline of GC include expanding our model-based approach to women and race/ethnicity subgroups by incorporating underlying differential risk factor trends to explore their combined effects on population-level cancer outcomes and disparities.

In conclusion, trends in modifiable risk factors explain a significant proportion of the decline of intestinal-type NCGA incidence in the US, and will contribute to future decline. While lower *H. pylori* infection rates have reduced cancer risk, other environmental factors may be equally important. Although past tobacco control efforts have hastened the decline, the full benefits will take several decades to be realized, and further discouragement of smoking and reduction of *H. pylori* infection should be priorities for GC control efforts.

## Supporting Information

Text S1 **Additional supporting information.**
(PDF)

## Abbreviations

GC: gastric cancer
NCGA: noncardia gastric adenocarcinoma
NHANES: National Health and Nutrition Examination Survey
NHIS: National Health Interview Survey
SEER: Surveillance, Epidemiology and End Results

## References

[1] Ferlay J, Bray F, Pisani P, Parkin DM (2004) GLOBOCAN 2002. cancer incidence, mortality and prevalence worldwide. IARC Cancer Base No. 5 Version 2.0. Lyon: IARCPress.

[2] Howlader N, Noone A, Krapcho M, Neyman N, Aminou R, et al (2012) SEER cancer statistics review, 1975–2009 (vintage 2009 populations). Bethesda (Maryland): National Cancer Institute. Available: http://seer.cancer.gov/csr/1975_2009_pops09/, based on November 2011 SEER data submission, posted to the SEER web site, April 2012.

[3] Howson CP, Hiyama T, Wynder EL (1986) The decline in gastric cancer: epidemiology of an unplanned triumph. Epidemiol Rev 8: 1–27. 3533579 10.1093/oxfordjournals.epirev.a036288

[4] IARC (1994) Schisotomes, liver flukes and helicobacter pylori. Monographs on the EVALuation of carcinogenic risks to humans number 61. Lyon: IARC Scientific Publishers. PMC7681621 7715068

[5] Blot WJ, Devesa SS, Kneller RW, Fraumeni JF Jr (1991) Rising incidence of adenocarcinoma of the esophagus and gastric cardia. JAMA 265: 1287–1289. 1995976

[6] Camargo MC, Anderson WF, King JB, Correa P, Thomas CC, et al (2011) Divergent trends for gastric cancer incidence by anatomical subsite in US adults. Gut 60: 1644–1649. 21613644 10.1136/gut.2010.236737 PMC3202077

[7] Surveillance, Epidemiology, and End Results (SEER) Program. SEER*Stat database: incidence - SEER 9 regs research data, Nov 2010 Sub (1973–2008) <Katrina/Rita Population Adjustment> - linked to county attributes - total U.S., 1969–2009 counties, National Cancer Institute, DCCPS, Surveillance Research Program, Cancer Statistics Branch, released April 2011, based on the November 2010 submission. Available: www.seer.cancer.gov.

[8] Correa P (1992) Human gastric carcinogenesis: a multistep and multifactorial process–first American Cancer Society Award lecture on cancer epidemiology and prevention. Cancer Res 52: 6735–6740. 1458460

[9] Kuipers EJ, Uyterlinde AM, Pena AS, Roosendaal R, Pals G, et al (1995) Long-term sequelae of Helicobacter pylori gastritis. Lancet 345: 1525–1528. 7791437 10.1016/s0140-6736(95)91084-0

[10] Helicobacter and Cancer Collaborative Group (2001) Gastric cancer and Helicobacter pylori: a combined analysis of 12 case control studies nested within prospective cohorts. Gut 49: 347–353. 11511555 10.1136/gut.49.3.347 PMC1728434

[11] Kato I, Vivas J, Plummer M, Lopez G, Peraza S, et al (2004) Environmental factors in Helicobacter pylori-related gastric precancerous lesions in Venezuela. Cancer Epidemiol Biomarkers Prev 13: 468–476. 15006925

[12] Russo A, Maconi G, Spinelli P, Felice GD, Eboli M, et al (2001) Effect of lifestyle, smoking, and diet on development of intestinal metaplasia in H. pylori-positive subjects. Am J Gastroenterol 96: 1402–1408. 11374674 10.1111/j.1572-0241.2001.03773.x

[13] Kneller RW, You WC, Chang YS, Liu WD, Zhang L, et al (1992) Cigarette smoking and other risk factors for progression of precancerous stomach lesions. J Natl Cancer Inst 84: 1261–1266. 1640486 10.1093/jnci/84.16.1261

[14] You WC, Zhang L, Gail MH, Chang YS, Liu WD, et al (2000) Gastric dysplasia and gastric cancer: Helicobacter pylori, serum vitamin C, and other risk factors. J Natl Cancer Inst 92: 1607–1612. 11018097 10.1093/jnci/92.19.1607

[15] Chao A, Thun MJ, Henley SJ, Jacobs EJ, McCullough ML, et al (2002) Cigarette smoking, use of other tobacco products and stomach cancer mortality in US adults: The Cancer Prevention Study II. Int J Cancer 101: 380–389. 12209964 10.1002/ijc.10614

[16] Kruszon-Moran D, McQuillan GM (2005) Seroprevalence of six infectious diseases among adults in the United States by race/ethnicity: data from the third national health and nutrition examination survey, 1988–94. Adv Data 1–9. 15771149

[17] McGinnis JM, Shopland D, Brown C (1987) Tobacco and health: trends in smoking and smokeless tobacco consumption in the United States. Annu Rev Public Health 8: 441–467. 3555529 10.1146/annurev.pu.08.050187.002301

[18] Schoenborn CA, Adams PE (2010) Health behaviors of adults: United States, 2005–2007. Vital Health Stat 10: 1–132. 20669609

[19] Surgeon General's Advisory Committee on Smoking and Health (1964) Smoking and health – report. Washington: U.S. Department of Health, Education and Welfare, Public Health Service, Center for Disease Control. PHS publication number 1103

[20] Lauren PA (1965) The two histological main types of gastric carcinoma: diffuse and so-called intestinal type carcinoma. Acta Path Microbiol Scand 64: 31. 14320675 10.1111/apm.1965.64.1.31

[21] Henson DE, Dittus C, Younes M, Nguyen H, Albores-Saavedra J (2004) Differential trends in the intestinal and diffuse types of gastric carcinoma in the United States, 1973–2000: increase in the signet ring cell type. Arch Pathol Lab Med 128: 765–770. 15214826 10.5858/2004-128-765-DTITIA

[22] Wu H, Rusiecki JA, Zhu K, Potter J, Devesa SS (2009) Stomach carcinoma incidence patterns in the United States by histologic type and anatomic site. Cancer Epidemiol Biomarkers Prev 18: 1945–1952. 19531677 10.1158/1055-9965.EPI-09-0250 PMC2786772

[23] Yeh JM, Kuntz KM, Ezzati M, Hur C, Kong CY, et al (2008) Development of an empirically calibrated model of gastric cancer in two high-risk countries. Cancer Epidemiol Biomarkers Prev 17: 1179–1187. 18483340 10.1158/1055-9965.EPI-07-2539

[24] Xia HH, Talley NJ (1997) Natural acquisition and spontaneous elimination of Helicobacter pylori infection: clinical implications. Am J Gastroenterol 92: 1780–1787. 9382036

[25] Gisbert JP (2005) The recurrence of Helicobacter pylori infection: incidence and variables influencing it. A critical review. Am J Gastroenterol 100: 2083–2099. 16128956 10.1111/j.1572-0241.2005.50043.x

[26] Anderson C, Burns D, Dodd K, Feuer E (2012) Chapter 2: birth-cohort-specific estimates of smoking behaviors for the U.S. population. Risk Analysis 32: S14–S24. 22882884 10.1111/j.1539-6924.2011.01703.x PMC4570501

[27] Rosenberg M, Feuer E, Yu B, Sun J, Henley S, et al (2012) Chapter 3: cohort life tables by smoking status, removing lung cancer as a cause of death. Risk Analysis 32: S25–S38. 22882890 10.1111/j.1539-6924.2011.01662.x PMC3594098

[28] Moolgavkar SH, Holford TR, Levy DT, Kong CY, Foy M, et al (2012) Impact of reduced tobacco smoking on lung cancer mortality in the United States during 1975–2000. J Natl Cancer Inst 104: 541–548. 22423009 10.1093/jnci/djs136 PMC3317881

[29] Day NE, Brown CC (1980) Multistage models and primary prevention of cancer. J Natl Cancer Inst 64: 977–989. 6929006

[30] Bell FC, Miller ML (2005) Life tables for the United States Social Security area 1900–2100. Baltimore (Maryland): Social Security Administration, Office of the Chief Actuary.

[31] Thun MJ, Myers DG, Day-Lally C, Namboodiri MM, Calle EE, et al.. (1997) Chapter 5: age and the exposure-response relationships between cigarette smoking and premature death in cancer prevention study II. Burns DM, Garfinkel L, Samet JM, editors. Smoking and tobacco control monograph number 8 Changes in cigarette-related disease risks and their implications for prevention and control. NIH publication – number 97-4213. Bethesda (Maryland): National Institutes of Health, National Cancer Institute.

[32] Day J (1996) Population projections of the United States by age, sex, race, and hispanic origin: 1995 to 2050. Washington (D.C.): U.S. Government Printing Office.

[33] U.S. Census Bureau, Population Division (2011) Population estimates. Washington (D.C.): U.S. Census Bureau.

[34] Fennerty MB, Emerson JC, Sampliner RE, McGee DL, Hixson LJ, et al (1992) Gastric intestinal metaplasia in ethnic groups in the southwestern United States. Cancer Epidemiol Biomarkers Prev 1: 293–296. 1303129

[35] Feuer EJ, Levy DT, McCarthy WJ (2012) Chapter 1: the impact of the reduction in tobacco smoking on U.S. Lung cancer mortality, 1975–2000: an introduction to the problem. Risk Anal 32 Suppl 1: S6–S13. 22882893 10.1111/j.1539-6924.2011.01745.x PMC4688905

[36] de Martel C, Ferlay J, Franceschi S, Vignat J, Bray F, et al (2012) Global burden of cancers attributable to infections in 2008: a review and synthetic analysis. Lancet Oncol 13: 607–615. 22575588 10.1016/S1470-2045(12)70137-7

[37] Hirayama T (1984) Epidemiology of stomach cancer in Japan. With special reference to the strategy for the primary prevention. Jpn J Clin Oncol 14: 159–168. 6737706

[38] World Cancer Research Fund/American Institute for Cancer Research (2007) Food, nutrition, physical activity, and the prevention of cancer: a global perspective. Washington (D.C.): American Institute for Cancer Research.

[39] Tsugane S (2005) Salt, salted food intake, and risk of gastric cancer: epidemiologic evidence. Cancer Sci 96: 1–6. 15649247 10.1111/j.1349-7006.2005.00006.x PMC11158463

[40] Shikata K, Kiyohara Y, Kubo M, Yonemoto K, Ninomiya T, et al (2006) A prospective study of dietary salt intake and gastric cancer incidence in a defined Japanese population: the Hisayama study. Int J Cancer 119: 196–201. 16450397 10.1002/ijc.21822

[41] Wroblewski LE, Peek RM Jr, Wilson KT (2010) Helicobacter pylori and gastric cancer: factors that modulate disease risk. Clin Microbiol Rev 23: 713–739. 20930071 10.1128/CMR.00011-10 PMC2952980

[42] Tuomilehto J, Geboers J, Joossens JV, Salonen JT, Tanskanen A (1984) Trends in stomach cancer and stroke in Finland. Comparison to northwest Europe and USA. Stroke 15: 823–828. 6474533 10.1161/01.str.15.5.823

[43] Peleteiro B, La Vecchia C, Lunet N (2011) The role of Helicobacter pylori infection in the web of gastric cancer causation. Eur J Cancer Prev 21: 118–125. 10.1097/CEJ.0b013e32834a7f66 21862926

[44] Sheen E, Triadafilopoulos G (2011) Adverse effects of long-term proton pump inhibitor therapy. Dig Dis Sci 56: 931–950. 21365243 10.1007/s10620-010-1560-3

[45] Anderson WF, Camargo MC, Fraumeni JF Jr, Correa P, Rosenberg PS, et al (2010) Age-specific trends in incidence of noncardia gastric cancer in US adults. JAMA 303: 1723–1728. 20442388 10.1001/jama.2010.496 PMC3142962

[46] Schlansky B, Sonnenberg A (2011) Epidemiology of noncardia gastric adenocarcinoma in the United States. Am J Gastroenterol 106: 1978–1985. 22008896 10.1038/ajg.2011.213

[47] Lau M, Le A, El-Serag HB (2006) Noncardia gastric adenocarcinoma remains an important and deadly cancer in the United States: secular trends in incidence and survival. Am J Gastroenterol 101: 2485–2492. 17029617 10.1111/j.1572-0241.2006.00778.x

[48] Edwards BK, Ward E, Kohler BA, Eheman C, Zauber AG, et al (2010) Annual report to the nation on the status of cancer, 1975–2006, featuring colorectal cancer trends and impact of interventions (risk factors, screening, and treatment) to reduce future rates. Cancer 116: 544–573. 19998273 10.1002/cncr.24760 PMC3619726

[49] Berry DA, Inoue L, Shen Y, Venier J, Cohen D, et al (2006) Modeling the impact of treatment and screening on U.S. breast cancer mortality: a Bayesian approach. J Natl Cancer Inst Monogr 30–36. 17032892 10.1093/jncimonographs/lgj006

[50] Ahmad A, Govil Y, Frank BB (2003) Gastric mucosa-associated lymphoid tissue lymphoma. Am J Gastroenterol 98: 975–986. 12809817 10.1111/j.1572-0241.2003.07424.x

[51] Burns DM, Lee LL, Gilpin B, Tolley HD, Vaughn J, et al.. (1997) Chapter 2: Cigarette smoking behavior in the United States. Burns DM, Garfinkel L, Samet JM, editors. Smoking and tobacco control monograph number 8. Changes in cigarette-related disease risks and their implications for prevention and control NIH publication – number 97-4213. Bethesda (Maryland): National Institutes of Health, National Cancer Institute.

[52] Lopez A, Collishaw NE, Piha T (1994) A descriptive model of the cigarette epidemic in developed countries. Tob Control 3: 242–247.

[53] Grad YH, Lipsitch M, Aiello AE (2012) Secular trends in Helicobacter pylori seroprevalence in adults in the United States: evidence for sustained race/ethnic disparities. Am J Epidemiol 175: 54–59. 22085628 10.1093/aje/kwr288 PMC3244610

[54] Mendez D, Warner KE (2004) Adult cigarette smoking prevalence: declining as expected (not as desired). Am J Public Health 94: 251–252. 14759934 10.2105/ajph.94.2.251 PMC1448235

[55] Smoking cessation during previous year among adults–United States, 1990 and 1991. MMWR Morb Mortal Wkly Rep 42: 504–507. 8515740

