# Supplementary material for: Contribution of H. pylori and Smoking Trends to US Incidence of Intestinal-Type Noncardia Gastric Adenocarcinoma: A Microsimulation Model
Source: PLoS Med. 2013 May 21;10(5):e1001451. doi: 10.1371/journal.pmed.1001451 (PMC3660292; doi:10.1371/journal.pmed.1001451)
Supplement: Text S1 — Additional supporting information. (PDF) [file pmed.1001451.s001.pdf]

Contribution of *H. pylori* and Smoking Trends to US Incidence of Intestinal-type Noncardia Gastric Adenocarcinoma: a Microsimulation Model

TEXT S1: ADDITIONAL SUPPORTING INFORMATION

Jennifer M. Yeh, PhD[1]

Chin Hur, MD, MPH[2]

Deb Schrag, MD, MPH[3]

Karen M. Kuntz, ScD[4]

Majid Ezzati, PhD[5]

Natasha Stout, PhD[6]

Zachary Ward, MPH[1]

Sue J. Goldie, MD, MPH[1]

From the [1]Center for Health Decision Science, Harvard School of Public Health, Boston, MA, USA; [2]Massachusetts General Hospital Institute for Technology Assessment, Boston, MA, USA; [3]Dana-Farber Cancer Institute, Harvard Medical School, Boston, MA, USA; [4]University of Minnesota School of Public Health, Minneapolis, MN, USA; [5]Imperial College of London, London, England; [6]Department of Population Medicine Harvard Medical School and Harvard Pilgrim Health Care Institute, Boston, MA.

ADDITIONAL DETAILS ON MODEL DEVELOPMENT AND CALIBRATION

Intestinal-type noncardia gastric adenocarcinoma (NCGA) microsimulation model

The population-based gastric cancer model simulates the natural history of intestinal-type NCGA. In a Monte Carlo simulation, individuals transition among health states one at a time and the detailed information for each individual is continuously tracked, allowing the natural history and course of disease to be conditional on that individual's risk factor profile. Specifically, events are simulated for a sequence of individuals using random numbers based on event probabilities (e.g., the probability of progressing from intestinal metaplasia to dysplasia). An individual's risk factors may be allowed to change each year to reflect changes in status.

Based on epidemiologic data [1], we assumed that precancerous lesions were already present in a subset of 20-years olds, with a proportion being greater among those infected with *H. pylori*. Because smoking initiation largely occurs between the ages of 15 and 30, we assumed smoking did not increase the presence of precancerous lesions at age 20. Individuals are simulated until they die or reach the final calendar year of analysis.

Smoking-specific background mortality

To reflect the higher risk of background mortality among smokers, we calculated smoking intensity-specific rates using age-specific birth cohort rates from the Berkeley Mortality Database (http://www.demog.berkeley.edu/~bmd/) and U.S. Social Security Administration [2], relative risk (RR) estimates (range = 1.1-3.8) based on the American Cancer Society Cancer Prevention Study II (CPS-II) [3] and the prevalence of smokers by intensity type at each year. We assumed that 1) for smokers ages 55 and younger, heavy smokers faced a 1.3 and 1.2 higher risk of background mortality compared to low and moderate smokers, respectively, 2) for individuals who quit smoking, background mortality risk remained elevated to intensity-specific levels for 5 years, and then declined afterwards to a constant relative risk for the remainder of his/her lifetime (RR = 1.5) [4], and 3) relative risks were constant across birth cohorts.

Natural history progression rates (identified via model calibration)

To infer unobservable natural history progression rates among precancerous lesions to invasive cancer, we used a likelihood-based calibration approach, previously described [5], to estimate natural history parameters and ensure model predictions are consistent with epidemiologic data. In short, based on the published literature, we established *a priori* plausible ranges for all model input parameters, including natural history parameters and relative risks associated with *H. pylori* and smoking [6,7,8,9,10,11,12,13,14,15,16,17,18,19,20,21,22,23] (see Table S1). We then specified calibration targets from epidemiologic studies and Surveillance, Epidemiology and End Results (SEER): age-specific intestinal metaplasia prevalence in 1990 (10-year age groups between ages 30 and 89), age-specific intestinal-type NCGA incidence for each year between 1978-1982 and 2004-2008 (5-year age groups between ages 35 and 85+ for a total of 10 years) and overall stage distribution for each year between 1978-1982 and 2004-2008 [24,25]. Identification of intestinal-type NCGA cases in SEER were based on the following criteria proposed by Lauren (1965) [26] and used by Henson et al. (2004) [27] and Wu et al. (2009) [28]: noncardia anatomical site (International Classification of Disease for Oncology (ICD-O) C16.1- C16.6, C16.8-C16.9) and intestinal histological subtype (M8010, M8140, M8211, M8144).

To explore the parameter space defined by the plausible ranges for our model parameters, we used a simulating annealing algorithm, a computationally feasible and time-efficient directed parameter search for microsimulation models [29]. Goodness-of-fit was defined based on the sum of log likelihoods for each outcome (assuming binomial distributions for all parameters). In addition, to identify parameter sets that fit both to our calibration targets and exhibited a decline in age-standardized intestinal-type NCGA incidence over time as observed in SEER, we included a slope variable to our goodness-of-fit, equal to the difference squared between the simulated and SEER slope between 1978-79 and 2007-08. We assumed that baseline disease progression rates remained constant across birth cohorts, although among birth cohorts, overall rates would vary due to birth-cohort specific risk factor patterns.

To reflect the uncertainty in disease natural history on our modeled outcomes, we conducted 500 independent simulating annealing searches to identify multiple parameter sets

that fit equally well to the calibration targets. Each simulating annealing search entailed 500 directed searches, each simulating 15.5 million individuals (7.5% of the population size for each birth cohort).

Among the 500 searches, we then identified 119 parameter sets with statistically similar fit to the best-fitting parameter set (α= 0.05). Among the 500 searches, we then identified 119 parameter sets with statistically similar fit to the best-fitting parameter set (α= 0.05). From these "good-fitting" parameter sets, we randomly selected a subset of 50 to reflect uncertainty in disease natural history and serve as natural history parameters for our scenario analyses (see Table 1 of the main text). For all modeled outcomes, we report the expected value (mean) and range (minimum, maximum) among the 50 parameter sets. As depicted in Figures S1-S3, the majority of modeled outcomes fell within the 95% confidence intervals of the calibration targets on intestinal metaplasia prevalence, cancer incidence and cancer stage distribution, though there was variability among the parameter sets.

Model validation

For model validation, we compared model outputs to data not used for either initial model parameterization or calibration. Prevalence of dysplasia (ranging 0.7% to 1.5% between 1978 and 2008) approximated published estimates in Western countries (0.5-3.8%) [30,31,32,33,34]. The modeled 10-year intestinal-type NCGA risk for a cohort of 65-year olds with dysplasia was 6.0% (range among 50 random parameter sets = 4.3-7.9%) which approximated estimates in the Netherlands (5.9%) [35]. Figure S4 depicts modeled 10-year cumulative at additional ages (1921-1925 birth cohort).

In addition, the relative risks of intestinal-type NCGA associated with *H. pylori* (4.7 vs. 5.9 [36]) or smoking (1.9 vs. 2.1 [37]) approximately published estimates. Relative risks stratified by both *H. pylori* and smoking status also approximated estimates from a population-based study on the combined impact on cancer incidence [38]: *H. pylori*-negative, current smoking (3.5 vs. 5.8 published estimate), *H. pylori*-positive, nonsmoking (4.6 vs. 6.9 published estimate), and *H. pylori*-positive, current smoking (14.1 vs. 11.4 published estimate).

ADDITIONAL RESULTS: AGE-SPECIFIC INTESTINAL-TYPE NCGA INCIDENCE

Modeled age-specific intestinal-type NCGA incidence suggest that rates have declined and are projected to continue to decline due to observed risk factor trends (see Figure S5 for base case scenario). Under the ‘no tobacco control’ scenario, as depicted in Figure S6, age-specific rates were also estimated to decline between 1978 and 2040.

SUPPORTING TABLES

Table S1. Plausible ranges for model calibration

Parameter	Plausible range	Reference
Natural history (monthly probabilities)		
Gastritis to atrophy	0.0001-0.001	[7,8,10,11,12,13,16,17]
Atrophy to intestinal metaplasia	0.0005-0.02	[11,15,16,17]
Intestinal metaplasia to dysplasia	0.0002-0.001	[11,14,16,18]
Dysplasia to preclinical cancer		[11,15,18]
Age 20-29	0-0.000005	
Age 30-39	0-0.00001	
Age 40-49	0.000004-0.000025	
Age 50-59	0.00001-0.00033	
Age 60-69	0.00012-0.00078	
Age 70-79	0.0003-0.00175	
Age 80-89	0.0007-0.00375	
Age 90+	0.0015-0.01	
Preclinical to clinical cancer	0.02-0.083	[19]
Dysplasia to intestinal metaplasia	0.0001-0.009	[9,11,14,16]
Intestinal metaplasia to atrophy	0.0001-0.009	[11,14,15,16]
Atrophy to gastritis	0.0003-0.01	[11,14,15,16]
Clinical cancer detection (monthly probabilities)		
Local	0.001-0.02	[20,21]
Regional	0.005-0.07	[20,21]
Distant	0.02-0.4	[20,21]
Risk factors on disease progression (relative risk)		
H. pylori		
Gastritis to atrophy	1.9-41.3	[6]
Smoking		[6,22,23]
Atrophy to intestinal metaplasia†		
<20 cigarettes per day	1-3.9	[22]
≥20 cigarettes per day	1.3-17	[22]
Former smoker	1-3.9	[22]
Intestinal metaplasia to dysplasia†		
<10 cigarettes per day	1-2.8	[23]
≥10 cigarettes per day	2.1-6.0	[23]
Heavy smoker	1-2.8	[23]
All other risk factors		
Gastritis to atrophy*	0-0.10	assumption

*Constant exponential rate (r) of decline per birth cohort as described in the following equation: (1-r)^t, where t = year of birth – 1901.

SUPPORTING FIGURES

Figure S1. Comparison of model output for 50 randomly selected good-fitting parameter sets to age-specific intestinal metaplasia targets. Bold lines = 95% confidence intervals, non-bold lines = model output**.**

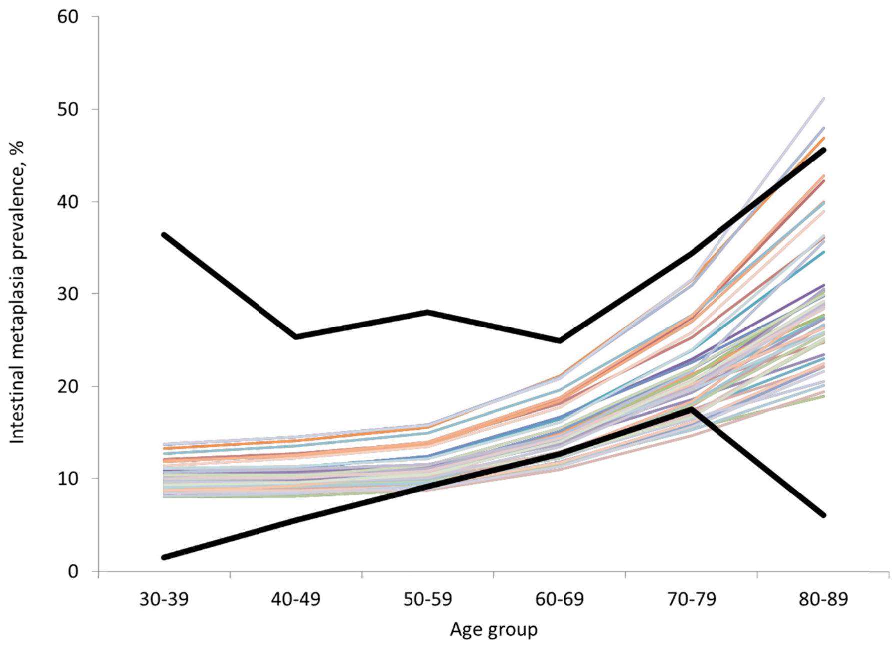

Figure S2. Comparison of model output for 50 randomly selected good-fitting parameter sets to age-specific intestinal-type NCGA incidence calibration targets. Panel A = 1978, Panel B = 2008**.** Bold lines = 95% confidence intervals, non-bold lines = model output.

A)

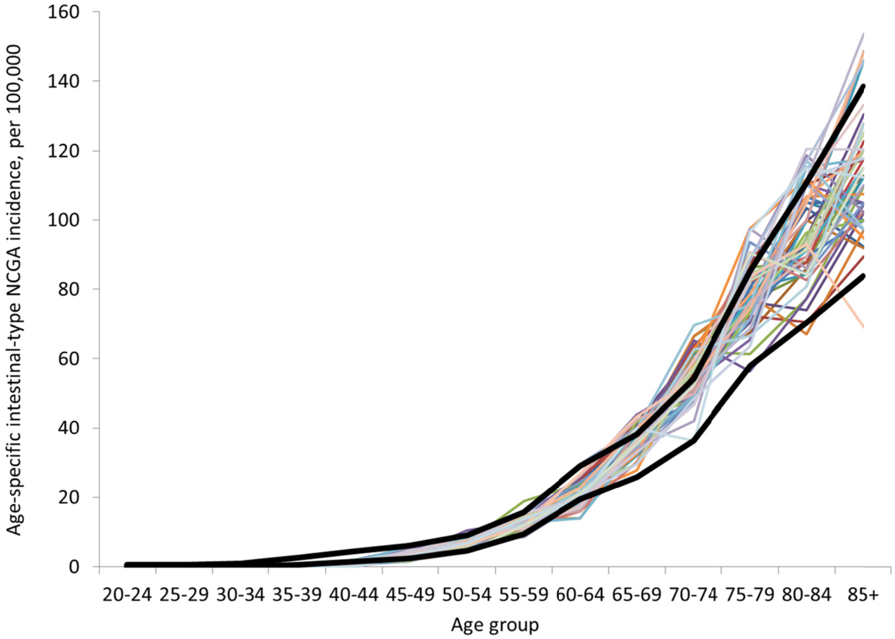

B)

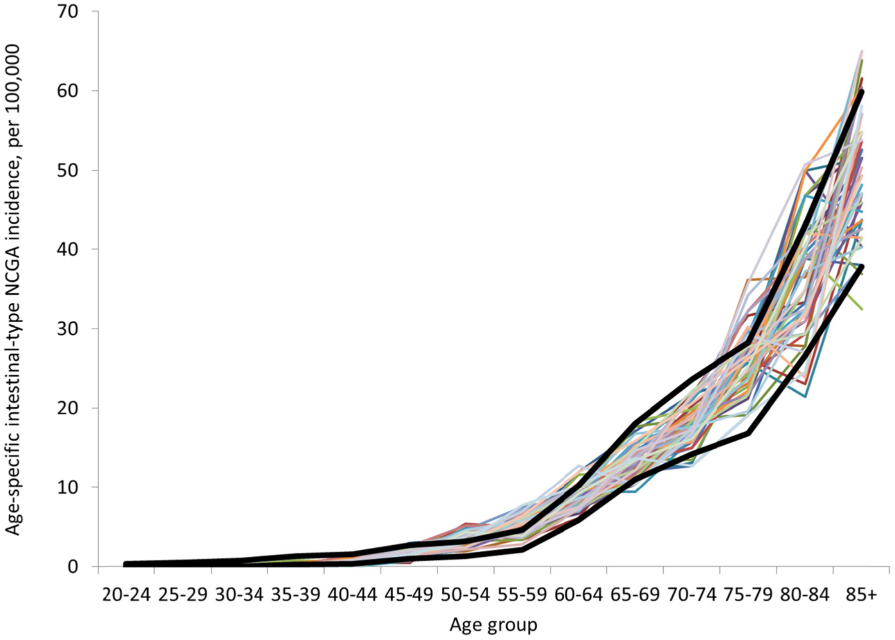

Figure S3. Comparison of model output for 50 randomly selected good-fitting parameter sets to stage-distribution calibration targets. Panel A = proportion of intestinal-type NCGAs diagnosed at localized stages, Panel B = proportion of intestinal-type NCGAs diagnosed at distant stages. Bold lines = 95% confidence intervals, non-bold lines = model output**.**

A)

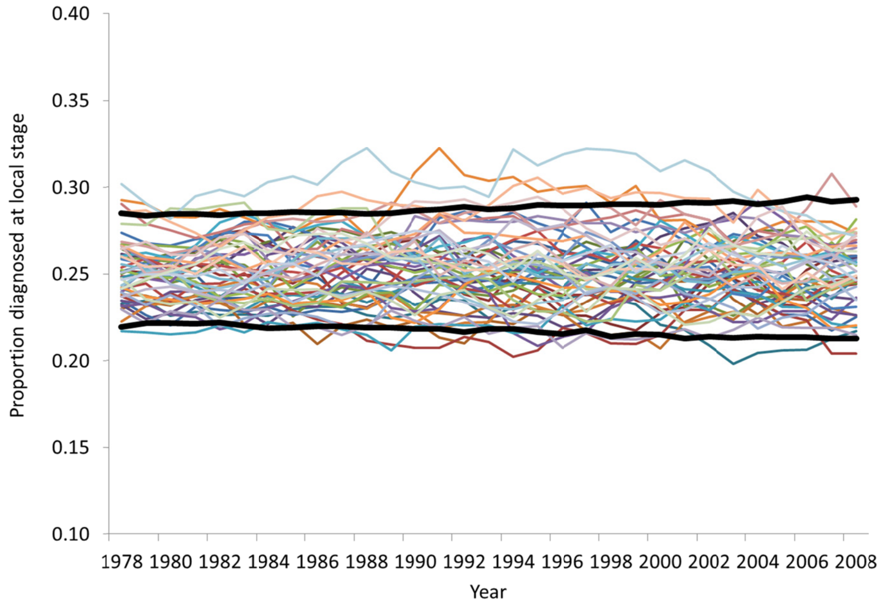

B)

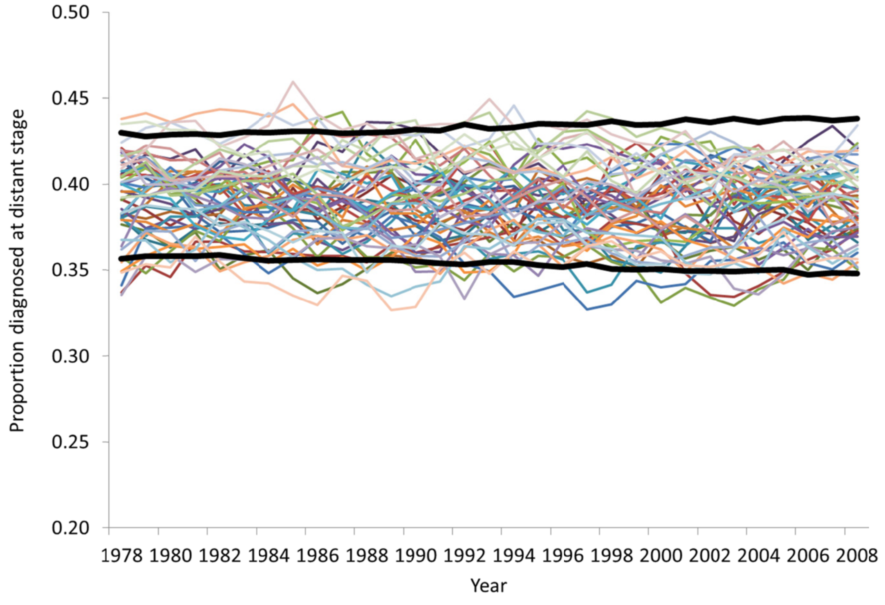

Figure S4. Modeled 10-year cumulative intestinal-type NCGA risk at select ages by precancerous lesions. Depicted are estimates for the mean (solid bars) and range (dotted lines) among 50 randomly selected good-fitting parameter sets. Atrophy = grey bars, intestinal metaplasia = light blue bars, and dysplasia = dark blue bars.

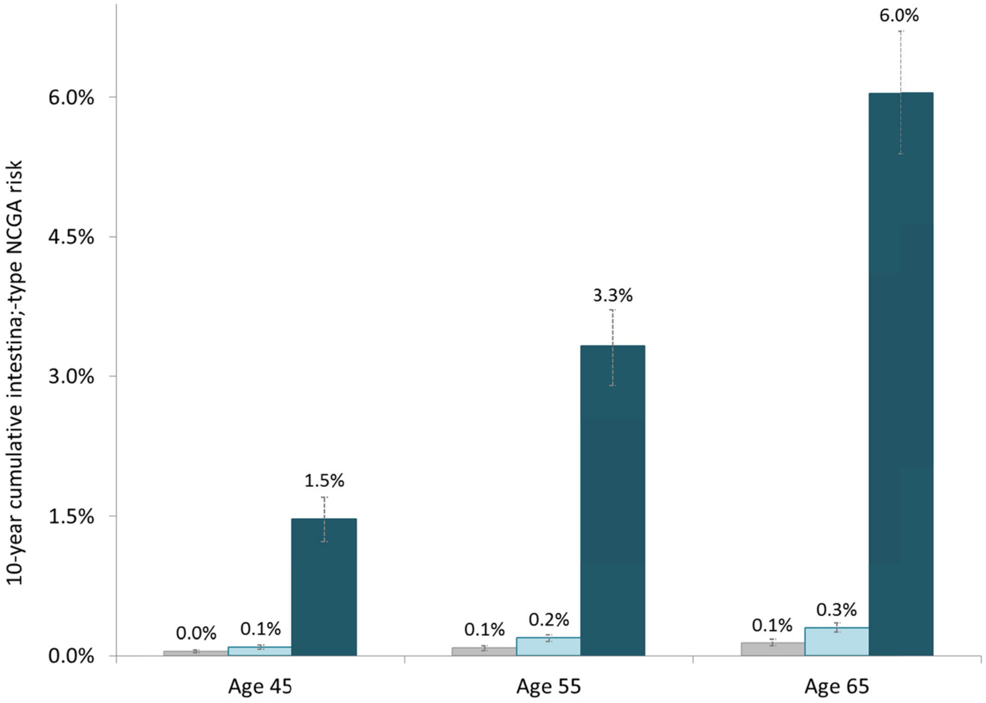

Figure S5. Modeled age-specific intestinal-type NCGA incidence for the base case scenario. Age-specific incidence for 5-year age groups between 1978 and 2040 are shown. Panel A depicts age groups between 20 and 44 years. Panel B depicts age groups between 45 and 85+ years.

A)

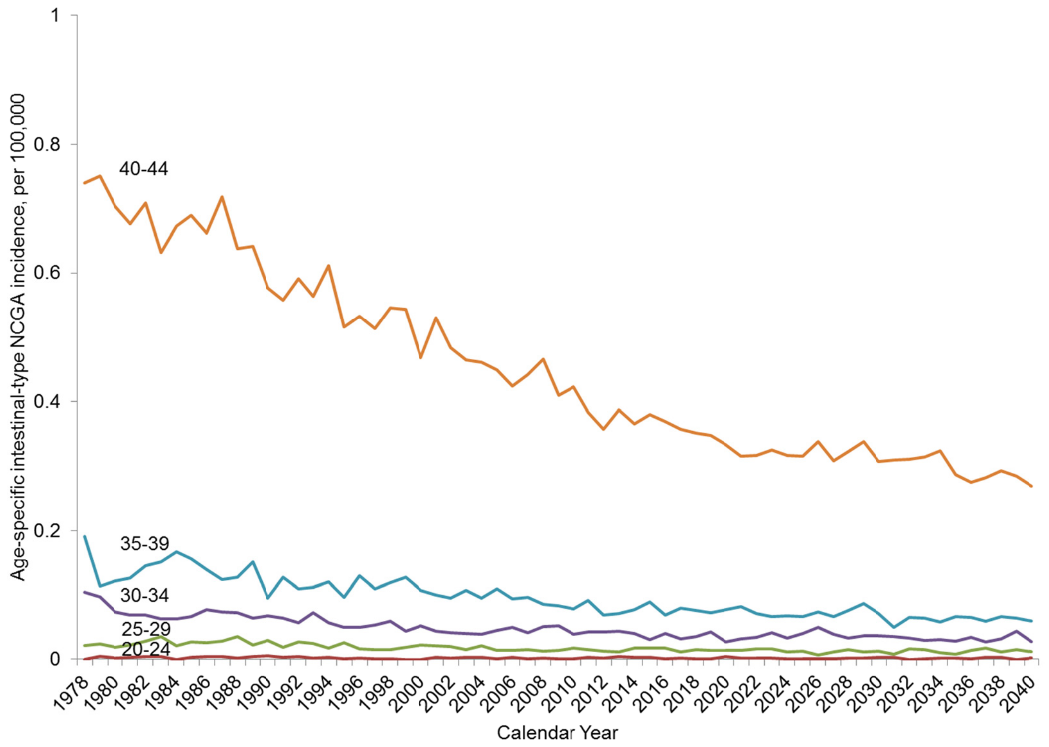

B)

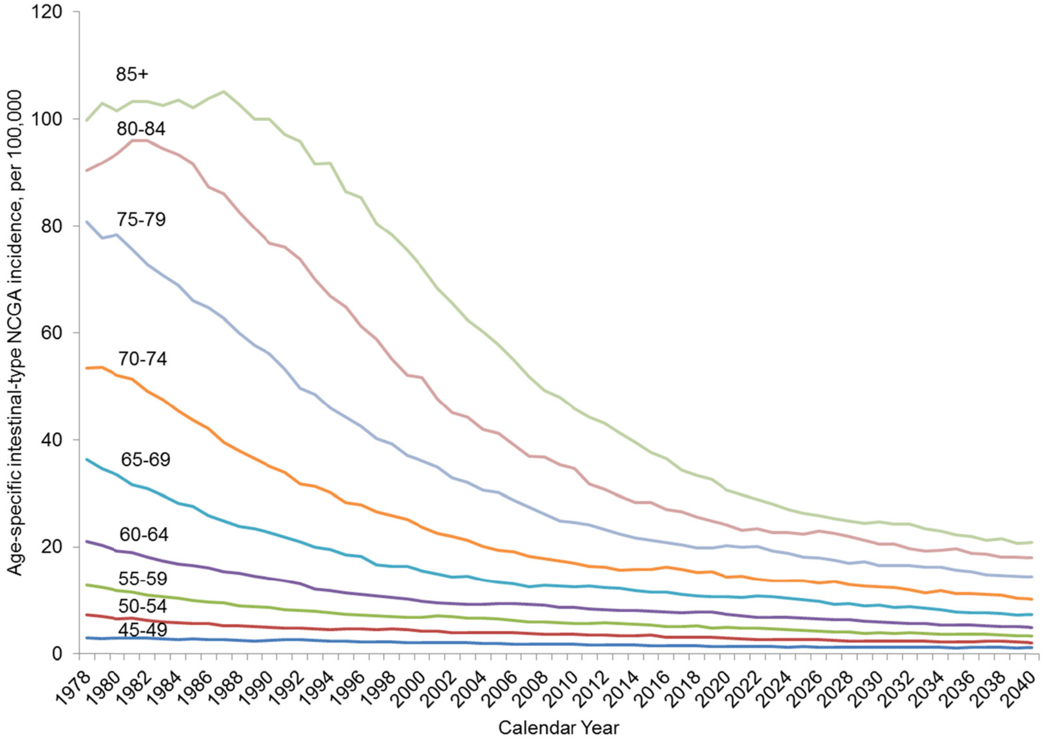

Figure S6. Modeled age-specific intestinal-type NCGA incidence for the 'no tobacco' scenario. Age-specific incidence for 5-year age groups between 1978 and 2040 are shown. Panel A depicts age groups between 20 and 44 years. Panel B depicts age groups between 45 and 85+ years

A)

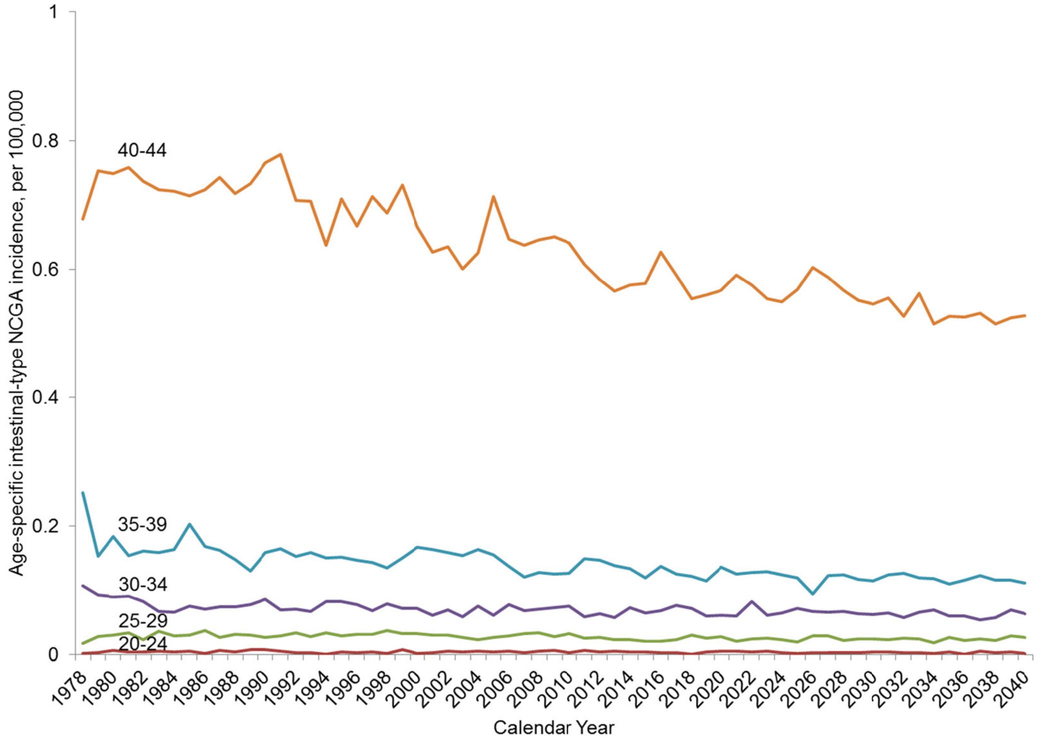

B)

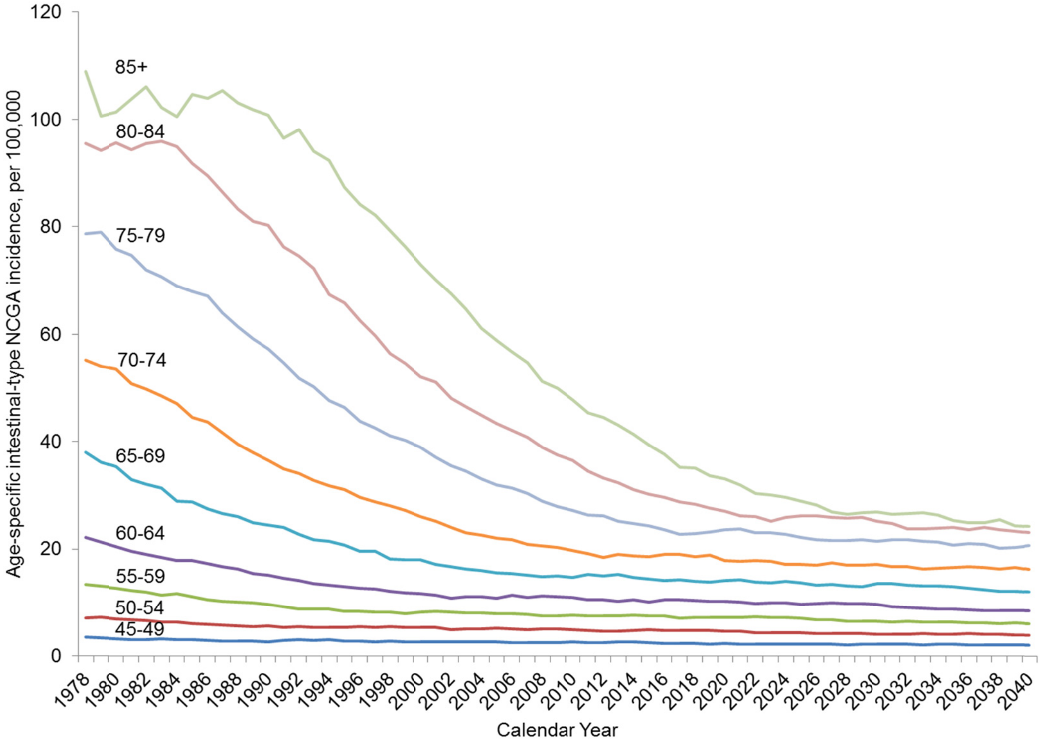

REFERENCES

1. Guarner J, Bartlett J, Whistler T, Pierce-Smith D, Owens M, et al. (2003) Can pre-neoplastic lesions be detected in gastric biopsies of children with Helicobacter pylori infection? J Pediatr Gastroenterol Nutr 37: 309-314.

2. Bell FC, Miller ML (2005) Life tables for the United States Social Security area 1900–2100. Baltimore, MD: Social Security Administration, Office of the Chief Actuary.

3. Thun MJ, Myers DG, Day-Lally C, Namboodiri MM, Calle EE, et al. (1997) Chapter 5: Age and the Exposure-Response Relationships Between Cigarette Smoking and Premature Death in Cancer Prevention Study II. In: Burns DM, Garfinkel L, Samet JM, editors. Smoking and Tobacco Control Monograph No 8 Changes in Cigarette-Related Disease Risks and Their Implications for Prevention and Control NIH publication -- no 97-4213. Bethesda, MD: National Institutes of Health, National Cancer Institute.

4. U.S. Department of Health and Human Services (1990) The Health Benefits of Smoking Cessation: A Report of the Surgeon General. DHHS Publication No. (CDC) 90-8416. U.S. Department of Health and Human Services. Public Health Service. Centers for Disease Control. Center for Chronic Disease Prevention and Health Promotion. Office of Smoking and Health.

5. Yeh JM, Kuntz KM, Ezzati M, Hur C, Kong CY, et al. (2008) Development of an empirically calibrated model of gastric cancer in two high-risk countries. Cancer Epidemiol Biomarkers Prev 17: 1179-1187.

6. Kuipers EJ, Uyterlinde AM, Pena AS, Roosendaal R, Pals G, et al. (1995) Long-term sequelae of Helicobacter pylori gastritis. Lancet 345: 1525-1528.

7. Gisbert JP (2005) The recurrence of Helicobacter pylori infection: incidence and variables influencing it. A critical review. Am J Gastroenterol 100: 2083-2099.

8. Xia HH, Talley NJ (1997) Natural acquisition and spontaneous elimination of Helicobacter pylori infection: clinical implications. Am J Gastroenterol 92: 1780-1787.

9. You WC, Blot WJ, Li JY, Chang YS, Jin ML, et al. (1993) Precancerous gastric lesions in a population at high risk of stomach cancer. Cancer Res 53: 1317-1321.

10. You WC, Zhang L, Gail MH, Li JY, Chang YS, et al. (1998) Precancerous lesions in two counties of China with contrasting gastric cancer risk. Int J Epidemiol 27: 945-948.

11. You WC, Li JY, Blot WJ, Chang YS, Jin ML, et al. (1999) Evolution of precancerous lesions in a rural Chinese population at high risk of gastric cancer. Int J Cancer 83: 615-619.

12. You WC, Zhang L, Gail MH, Chang YS, Liu WD, et al. (2000) Gastric dysplasia and gastric cancer: Helicobacter pylori, serum vitamin C, and other risk factors. J Natl Cancer Inst 92: 1607-1612.

13. Correa P, Haenszel W, Cuello C, Zavala D, Fontham E, et al. (1990) Gastric precancerous process in a high risk population: cohort follow-up. Cancer Res 50: 4737-4740.

14. Correa P, Haenszel W, Cuello C, Zavala D, Fontham E, et al. (1990) Gastric precancerous process in a high risk population: cross-sectional studies. Cancer Res 50: 4731-4736.

15. Correa P, Fontham ET, Bravo JC, Bravo LE, Ruiz B, et al. (2000) Chemoprevention of gastric dysplasia: randomized trial of antioxidant supplements and anti-helicobacter pylori therapy. J Natl Cancer Inst 92: 1881-1888.

16. Plummer M, Vivas J, Lopez G, Bravo JC, Peraza S, et al. (2007) Chemoprevention of precancerous gastric lesions with antioxidant vitamin supplementation: a randomized trial in a high-risk population. J Natl Cancer Inst 99: 137-146.

17. Gonzalez CA, Pardo ML, Liso JM, Alonso P, Bonet C, et al. (2010) Gastric cancer occurrence in preneoplastic lesions: a long-term follow-up in a high-risk area in Spain. Int J Cancer 127: 2654-2660.

18. Tava F, Luinetti O, Ghigna MR, Alvisi C, Perego M, et al. (2006) Type or extension of intestinal metaplasia and immature/atypical "indefinite-for-dysplasia" lesions as predictors of gastric neoplasia. Hum Pathol 37: 1489-1497.

19. Tsukuma H, Oshima A, Narahara H, Morii T (2000) Natural history of early gastric cancer: a non-concurrent, long term, follow up study. Gut 47: 618-621.

20. Craanen ME, Dekker W, Ferwerda J, Blok P, Tytgat GN (1991) Early gastric cancer: a clinicopathologic study. J Clin Gastroenterol 13: 274-283.

21. Fielding JW, Ellis DJ, Jones BG, Paterson J, Powell DJ, et al. (1980) Natural history of "early" gastric cancer: results of a 10-year regional survey. Br Med J 281: 965-967.

22. Russo A, Maconi G, Spinelli P, Felice GD, Eboli M, et al. (2001) Effect of lifestyle, smoking, and diet on development of intestinal metaplasia in H. pylori-positive subjects. Am J Gastroenterol 96: 1402-1408.

23. Kato I, Vivas J, Plummer M, Lopez G, Peraza S, et al. (2004) Environmental factors in Helicobacter pylori-related gastric precancerous lesions in Venezuela. Cancer Epidemiol Biomarkers Prev 13: 468-476.

24. Fennerty MB, Emerson JC, Sampliner RE, McGee DL, Hixson LJ, et al. (1992) Gastric intestinal metaplasia in ethnic groups in the southwestern United States. Cancer Epidemiol Biomarkers Prev 1: 293-296.

25. Surveillance, Epidemiology, and End Results (SEER) Program (www.seer.cancer.gov) SEER*Stat Database: Incidence - SEER 9 Regs Research Data, Nov 2010 Sub (1973-2008) <Katrina/Rita Population Adjustment> - Linked To County Attributes - Total U.S., 1969-2009 Counties, National Cancer Institute, DCCPS, Surveillance Research Program, Cancer Statistics Branch, released April 2011, based on the November 2010 submission.

26. Lauren PA (1965) The two histological main types of gastric carcinoma: diffuse and so-called intestinal type carcinoma. Acta Path Microbiol Scand 64: 31.

27. Henson DE, Dittus C, Younes M, Nguyen H, Albores-Saavedra J (2004) Differential trends in the intestinal and diffuse types of gastric carcinoma in the United States, 1973-2000: increase in the signet ring cell type. Arch Pathol Lab Med 128: 765-770.

28. Wu H, Rusiecki JA, Zhu K, Potter J, Devesa SS (2009) Stomach carcinoma incidence patterns in the United States by histologic type and anatomic site. Cancer Epidemiol Biomarkers Prev 18: 1945-1952.

29. Kong CY, McMahon PM, Gazelle GS (2009) Calibration of disease simulation model using an engineering approach. Value Health 12: 521-529.

30. Lauwers GY, Srivastava A (2007) Gastric preneoplastic lesions and epithelial dysplasia. Gastroenterol Clin North Am 36: 813-829, vi.

31. Bearzi I, Brancorsini D, Santinelli A, Rezai B, Mannello B, et al. (1994) Gastric dysplasia: a ten-year follow-up study. Pathol Res Pract 190: 61-68.

32. Camilleri JP, Potet F, Amat C, Molas G (1984) Gastric muscosal dysplasia: Preliminary results of a prospective study of patients followed for periods of up to six years. In: Ming SC, editor. Precursors of Gastric Cancer. New York: Praeger

33. Farinati F, Rugge M, Di Mario F, Valiante F, Baffa R (1993) Early and advanced gastric cancer in the follow-up of moderate and severe gastric dysplasia patients. A prospective study. I.G.G.E.D.--Interdisciplinary Group on Gastric Epithelial Dysplasia. Endoscopy 25: 261-264.

34. Koch HK, Oehlert M, Oehlert W (1990) An evaluation of gastric dysplasia in the years 1986 and 1987. Pathol Res Pract 186: 80-84.

35. de Vries AC, van Grieken NC, Looman CW, Casparie MK, de Vries E, et al. (2008) Gastric cancer risk in patients with premalignant gastric lesions: a nationwide cohort study in the Netherlands. Gastroenterology 134: 945-952.

36. Helicobacter and Cancer Collaborative Group (2001) Gastric cancer and Helicobacter pylori: a combined analysis of 12 case control studies nested within prospective cohorts. Gut 49: 347-353.

37. Ezzati M, Henley SJ, Lopez AD, Thun MJ (2005) Role of smoking in global and regional cancer epidemiology: current patterns and data needs. Int J Cancer 116: 963-971.

38. Shikata K, Doi Y, Yonemoto K, Arima H, Ninomiya T, et al. (2008) Population-based prospective study of the combined influence of cigarette smoking and Helicobacter pylori infection on gastric cancer incidence: the Hisayama Study. Am J Epidemiol 168: 1409-1415.
